# Severe Acute Respiratory Syndrome Coronavirus Envelope Protein Ion Channel Activity Promotes Virus Fitness and Pathogenesis

**DOI:** 10.1371/journal.ppat.1004077

**Published:** 2014-05-01

**Authors:** Jose L. Nieto-Torres, Marta L. DeDiego, Carmina Verdiá-Báguena, Jose M. Jimenez-Guardeño, Jose A. Regla-Nava, Raul Fernandez-Delgado, Carlos Castaño-Rodriguez, Antonio Alcaraz, Jaume Torres, Vicente M. Aguilella, Luis Enjuanes

**Affiliations:** 1 Department of Molecular and Cell Biology, Centro Nacional de Biotecnología (CNB-CSIC), Campus Universidad Autónoma de Madrid, Madrid, Spain; 2 Department of Physics, Laboratory of Molecular Biophysics. Universitat Jaume I, Castellón, Spain; 3 School of Biological Sciences, Division of Structural and Computational Biology, Nanyang Technological University, Singapore, Singapore; Vanderbilt University, United States of America

## Abstract

Deletion of Severe Acute Respiratory Syndrome Coronavirus (SARS-CoV) envelope (E) gene attenuates the virus. E gene encodes a small multifunctional protein that possesses ion channel (IC) activity, an important function in virus-host interaction. To test the contribution of E protein IC activity in virus pathogenesis, two recombinant mouse-adapted SARS-CoVs, each containing one single amino acid mutation that suppressed ion conductivity, were engineered. After serial infections, mutant viruses, in general, incorporated compensatory mutations within E gene that rendered active ion channels. Furthermore, IC activity conferred better fitness in competition assays, suggesting that ion conductivity represents an advantage for the virus. Interestingly, mice infected with viruses displaying E protein IC activity, either with the wild-type E protein sequence or with the revertants that restored ion transport, rapidly lost weight and died. In contrast, mice infected with mutants lacking IC activity, which did not incorporate mutations within E gene during the experiment, recovered from disease and most survived. Knocking down E protein IC activity did not significantly affect virus growth in infected mice but decreased edema accumulation, the major determinant of acute respiratory distress syndrome (ARDS) leading to death. Reduced edema correlated with lung epithelia integrity and proper localization of Na^+^/K^+^ ATPase, which participates in edema resolution. Levels of inflammasome-activated IL-1β were reduced in the lung airways of the animals infected with viruses lacking E protein IC activity, indicating that E protein IC function is required for inflammasome activation. Reduction of IL-1β was accompanied by diminished amounts of TNF and IL-6 in the absence of E protein ion conductivity. All these key cytokines promote the progression of lung damage and ARDS pathology. In conclusion, E protein IC activity represents a new determinant for SARS-CoV virulence.

## Author Summary

Several highly pathogenic viruses encode small transmembrane proteins with ion-conduction properties named viroporins. Viroporins are generally involved in virus production and maturation processes, which many times are achieved by altering the ion homeostasis of cell organelles. Cells have evolved mechanisms to sense these imbalances in ion concentrations as a danger signal, and consequently trigger the innate immune system. Recently, it has been demonstrated that viroporins are inducers of cytosolic macromolecular complexes named inflammasomes that trigger the activation of key inflammatory cytokines such as IL-1β. The repercussions of this system in viral pathogenesis or disease outcome are currently being explored. SARS-CoV infection induces an uncontrolled inflammatory response leading to pulmonary damage, edema accumulation, severe hypoxemia and eventually death. In this study, we report that SARS-CoV E protein ion channel activity is a determinant of virulence, as the elimination of this function attenuated the virus, reducing the harmful inflammatory cytokine burst produced after infection, in which inflammasome activation plays a critical role. This led to less pulmonary damage and to disease resolution. These novel findings may be of relevance for other viral infections and can possibly be translated in order to find therapies for their associated diseases.

## Introduction

Coronaviruses (CoVs) are vertebrate pathogens that cause severe diseases in a wide range of animals and infections in humans that until recently were limited to common colds [1]. Nevertheless, by the end of 2002, a novel coronavirus causing the severe acute respiratory syndrome (SARS-CoV) emerged in China and rapidly spread worldwide causing around 8000 infections leading to death in 10% of the cases [2], [3]. Since then, CoVs surveillance programs were intensified, and two additional human coronaviruses, already circulating in the human population, were identified as the causative agents of several cases of pneumonia and bronchiolitis (HCoV-HKU1 and HCoV-NL63) [4]. Furthermore, in 2012 a novel coronavirus infecting humans, the Middle East Respiratory Syndrome Coronavirus (MERS-CoV) appeared in Saudi Arabia and disseminated to nine additional countries [5], [6]. To date, 182 cases of MERS-CoV have been reported, which has led to 79 fatalities (http://www.who.int). Clinical presentation of infected individuals involves acute pneumonia, sometimes accompanied by renal disease [7]. CoVs similar to SARS-CoV and MERS-CoV have also been isolated from bats widely distributed throughout the world [8]–[13], which represents a potential reservoir for outbreaks of novel zoonoses into humans. Therefore, understanding the virulence mechanisms of these pathogens, will allow the development of effective therapies in order to prevent and control future outbreaks.

SARS-CoV is an enveloped virus containing a positive sense RNA genome of 29.7 kb, one of the largest viral RNA genomes known. The genome encodes a viral replicase involved in the synthesis of new genomes and in the generation of a nested set of subgenomic messenger RNAs, encoding both structural proteins present in all CoVs: Spike (S), Envelope (E), Membrane (M) and Nucleoprotein (N), and a group of proteins specific for SARS-CoV: 3a, 3b, 6, 7a, 7b, 8a, 8b, and 9b [14].

SARS-CoV E protein is a 76-amino acid transmembrane protein actively synthesized during viral infection, that mainly localizes at the ERGIC region of the cell, where virus budding and morphogenesis take place [15]–[18]. Different requirements of E protein during the virus cycle have been described among CoVs. Elimination of E gene in transmissible gastroenteritis coronavirus (TGEV) or MERS-CoV leads to a replication-competent propagation-deficient phenotype [19]–[21]. In contrast, deletion of E gene from mouse hepatitis virus (MHV) or SARS-CoV does not abolish virus production, although viral titers are significantly reduced by 1000 to 20-fold, respectively [16], [22]. Interestingly, E gene deleted SARS-CoV (SARS-CoV-ΔE) was attenuated in three animal models, and confers protection against challenge with parental virus in immunized hamsters, and in young or aged mice, representing a promising vaccine candidate [16], [23]–[27]. Cells infected with SARS-CoV-ΔE show increased stress and apoptotic markers compared to wild type virus, perhaps resulting in a decreased productivity of infection [28]. Additionally, elimination of the E gene diminishes inflammation induced by SARS-CoV through the NF-κB pathway [27].

Remarkably, SARS-CoV E protein was found to self-interact forming a pentameric structure that delimits an ion conductive pore, which may play a role in virus-host interaction [29]–[32]. E protein ion conductivity was also confirmed for a set of CoVs from different genera [33]. The ion channel (IC) activity of SARS-CoV E protein was mapped within the transmembrane domain of the protein by using synthetic peptides [31], [34], [35]. Recent studies determined that both ion conductance and selectivity of E protein ion channel were highly controlled by the charge of the lipid membranes in which the pores were assembled. This suggests that lipid head-groups are components of the channel structure facing the lumen of the pore, a novel concept for CoV E protein ion channel [34], [36]. Chemically synthesized SARS-CoV E protein showed slight preference for cations over anions when reconstituted in lipids that mimicked both charge and composition of ERGIC membranes, and displayed no specific selectivity for a particular cation [34], [36]. In addition, point mutations that suppressed SARS-CoV E protein IC activity (N15A and V25F) have been identified and confirmed [34], [35].

Several reports have analyzed the relevance of CoV E protein transmembrane domain, which contains ion-conduction properties, in virus maturation and production. Insertion of alanine residues within the transmembrane domain of MHV E protein rendered crippled viruses that evolutionary reverted to restore a proper structure of the alpha helix within the transmembrane domain [37]. Interchanging the genus β CoV MHV E protein transmembrane domain by those of CoVs from different genera revealed that only domains belonging to genus β, and γ, but not α, functionally replaced MHV E transmembrane domain in terms of viral production. It was speculated that this effect was a consequence of the possible different ion selectivity of these domains [38]. Replacement of genus γ CoV infectious bronchitis virus (IBV) E protein transmembrane domain, which displays IC activity, for vesicular stomatitis virus (VSV) G protein transmembrane domain lacking this function, interfered with an efficient trafficking and release of the viral progeny in the infected cells [39]. In contrast, mutation of threonine at position 16 to alanine, which is the amino acid change predicted to inhibit IC activity in IBV E protein did not affect virus-like particles formation, suggesting a multifunctional role of E protein [40].

Besides the E protein, SARS-CoV encodes two other ion-conducting proteins, 3a and 8a [41], [42]. In a related virus, human coronavirus 229E (HCoV-229E), novel IC activity has been described within the 4a protein [43]. The abundance and conservation of IC activity suggests an importance of influencing ion homeostasis within cells during the CoV infection cycle.

Modulation of the cellular ion balance seems to be a common issue for viruses, as a growing list of viroporins are being identified, especially within RNA viruses [44]. Highly pathogenic human viruses such as influenza A virus, human immunodeficiency virus (HIV), hepatitis C virus (HCV) and several picornaviruses, among others, encode at least one viroporin [45]–[49]. Viroporins have been involved in virus entry, trafficking, morphogenesis, maturation and even virulence [50]–[53]. Influenza virus M2 is essential for viral RNA release from infections virions within the endosome into the cell cytoplasm [45] and also for raising the pH at the trans-Golgi network lumen, which prevents premature activation of hemaglutinin, which may render non-infectious virions [54]. Similarly, HCV p7 protein equilibrates the pH at the Golgi apparatus, protecting acid-sensitive intracellular virions [51]. Coxsackievirus 2B protein alters Golgi and endoplasmic reticulum (ER) Ca^2+^ and H^+^ concentrations, which in turn delay protein transport through the secretory pathway facilitating virus assembly and preventing major histocompatibility complex (MHC) molecules from reaching the cell surface [48], [55], [56]. A recent finding described that influenza M2 protein IC activity triggers NOD-like receptor family, pyrin domain containing 3 (NLRP3) inflammasome activation [52]. Furthermore, mutant versions of M2 protein that conduct Na^+^ and K^+^ ions apart from H^+^ ions more strongly elicited the inflammasome response [52]. This novel mechanism of immune system activation has also been proven for other viroporins [53], [57]–[59].

Viral proteins with IC activity impact different aspects of the virus life cycle, however, the involvement of their IC activity in pathogenesis remain to be further explored. Previous findings demonstrated that SARS-CoV E protein is a virulence determinant. In this manuscript we analyze the contribution of E protein IC activity in pathogenesis. Two recombinant viruses, each one containing a single point mutation suppressing IC activity, were generated by reverse genetics. Mutant viruses showed a tendency to evolve and restore E protein IC architecture and activity after serial infections, and viruses with deficient IC activity were outcompeted by those displaying this function after co-infections. This highlights the importance of IC activity in virus fitness. Interestingly, infection of mice with a set of viruses lacking or displaying E protein IC activity, revealed that the activation of inflammasome pathway, and the exacerbated inflammatory response induced by SARS-CoV was decreased in infections by on channel deficient viruses. In addition, less lung damage and proper localization of Na^+^/K^+^ ATPase within epithelia, which prevents edema accumulation, was detected for the mice infected with the viruses lacking E protein IC activity. As a consequence, increased survival of the infected animals was observed when E protein ion conductivity was absent. Therefore, E protein IC activity is required for inflammasome activation and a novel determinant for the virulence of highly pathogenic SARS-CoV.

## Results

### SARS-CoV E protein IC activity is not essential for virus production in cell culture

**Figure 1:**
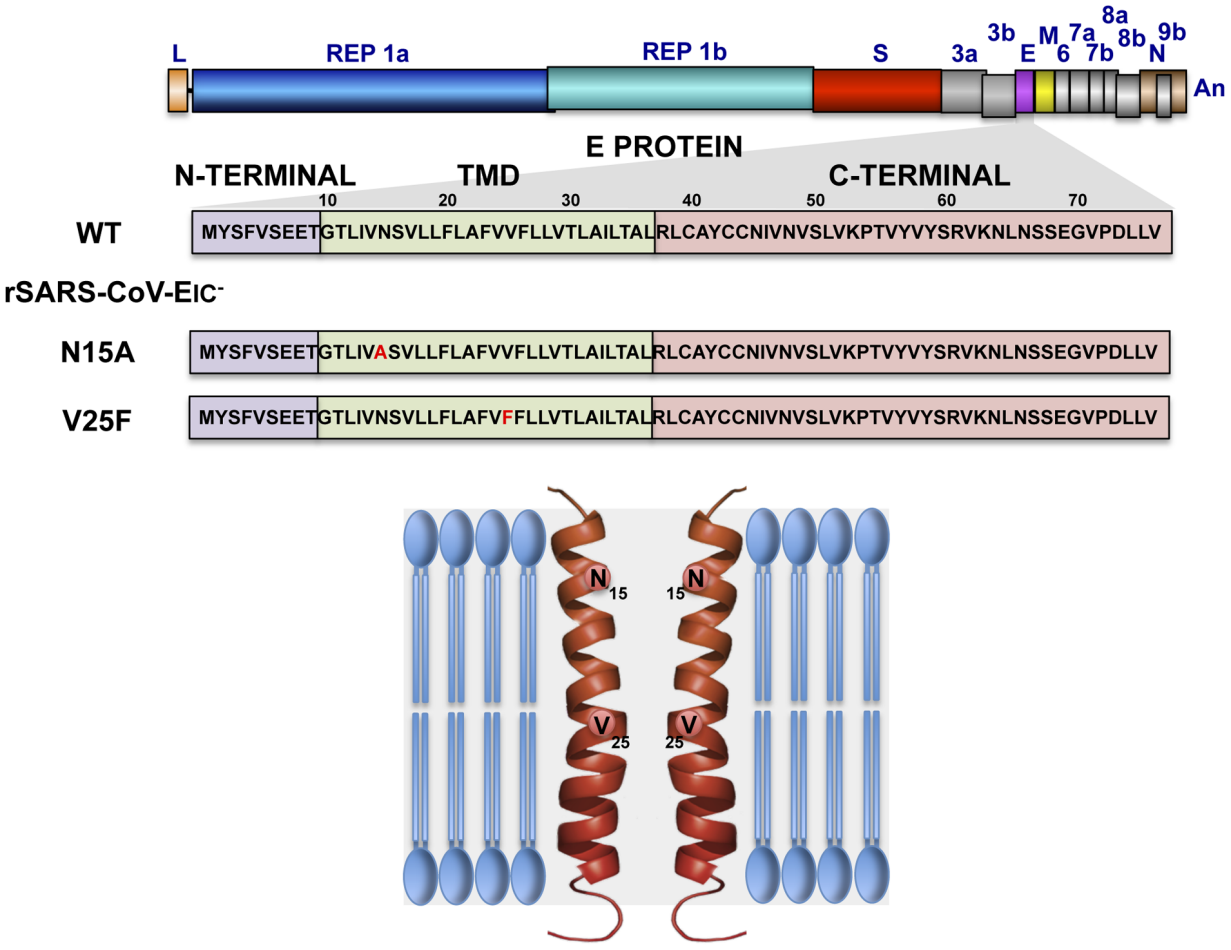
Engineering of rSARS-CoVs lacking E protein ion channel (IC) activity. SARS-CoV genome is represented at the top, and the region expanded shows wild type SARS-CoV E protein sequence (wt) and its different domains: amino terminal (N-terminal), transmembrane (TMD) and carboxy terminal (C-terminal). To generate viruses lacking E protein ion channel activity (rSARS-CoV-EIC^−^) the amino acid changes N15A or V25F were introduced within viral genome to generate two recombinant viruses. The positions of the mutated residues within the transmembrane domain of a simplified E protein oligomer inserted in a lipid membrane are shown at the bottom.

**Figure 2:**
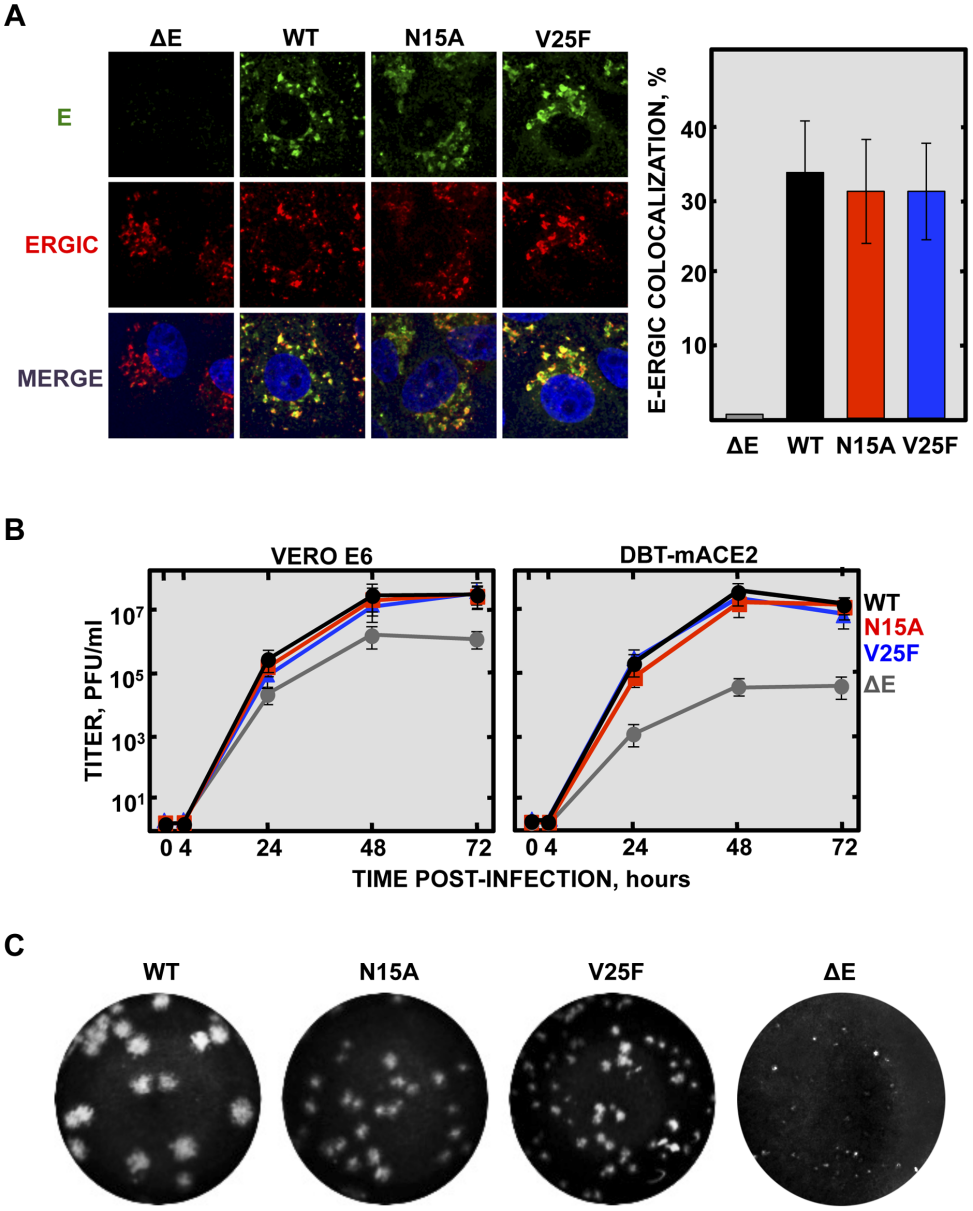
Subcellular localization of rSARS-CoV-EIC^−^ E proteins, growth kinetics and plaque size. (**A**) Vero E6 cells were infected either with the mutant viruses (N15A and V25F), the parental virus (wt) or a virus lacking E gene (ΔE) at an MOI of 0.3, fixed at 24 hpi and E protein (green) and ERGIC (red) were labeled with specific antibodies. Nuclei were stained with DAPI (blue). Original magnification was 126×. Right graphic on the panel represents the percentage of colocalization between E protein and ERGIC, calculated with Leica LAS AF v2.6.0 software. (**B**) Vero E6 and DBT-mACE2 cells were infected at an MOI of 0.001 with mutant viruses lacking IC activity (N15A and V25F), the parental virus (wt) or a virus lacking E gene (ΔE), and viral progeny was titrated at the indicated times post-infection. Error bars represent the standard deviation of three independent experiments. (**C**) Plaque morphology of the parental, the mutant viruses N15A and V25F and a ΔE virus.

Deletion of SARS-CoV E gene resulted in a virus that was attenuated in three animal models, as we have previously shown [16], [23], [24], [26], [27]. E gene codes for the small multifunctional E protein, which displays IC activity [31], [34]–[36]. To specifically test the relevance of IC activity in virus virulence, residues involved in E protein ion conductance were firstly identified. To this end a set of synthetic peptides representing the transmembrane domain of E protein were evaluated for their IC activity. These peptides contained point mutations that affect different conserved residues, or residues predicted to face the lumen of the channel pore [34]. Mutations N15A and V25F within the transmembrane domain of E protein completely disrupted IC activity [34], [35]. Accordingly, two recombinant viruses containing each of these two changes in the E gene, rSARS-CoV-E-N15A (N15A) and rSARS-CoV-E-V25F (V25F), were engineered (**Fig. 1**). A SARS-CoV with a mouse adapted (MA15) genetic background [27], [60] was used to generate these viruses, as infection of mice with SARS-CoV MA15 accurately reproduces the symptoms of human disease [27], [60]. The mutant viruses were efficiently rescued, cloned by three rounds of plaque purification, and their sequence was confirmed (data not shown). To test whether the introduced mutations may alter E protein subcellular localization affecting other functions of the protein, Vero E6 cells were infected with the wt virus, the viruses lacking IC activity (N15A and V25F) or a virus missing E gene (ΔE) as a control. Immunofluorescence analysis showed similar colocalization patterns of E protein and ERGIC, the subcellular compartment where E protein mainly accumulates during infection, for both the wt and the mutant viruses (**Fig. 2A**), indicating that other functions of E protein associated with its localization are most likely not affected.

Deletions or mutations within the E gene of several CoVs sometimes led to crippled viruses or to lower virus yields [16], [20]–[22], [37], [39]. To test whether inhibition of E protein IC activity affects virus production, growth kinetics were performed in the monkey Vero E6 and mouse DBT-mACE2 cells [61]. Minor differences in growth rates were observed between the parental virus (wt), that contains E protein IC activity, and the mutant viruses that lack E protein IC activity (**Fig. 2B**), indicating that this function was not essential for virus growth in cell culture. More striking differences in plaque phenotypes were observed. Mutant viruses lacking E protein IC activity, apparently formed smaller plaques than wt virus, and V25F virus plaques were smaller than N15A virus (**Fig. 2C**). A possible explanation for all these data could be that infection foci productivity and area may be quite similar regardless of E protein IC activity, as determined by viral titration, but higher cytopathic effect may be induced when E protein IC is present, rendering bigger plaques. Elimination of full-length E protein induced more severe growth defects (**Fig. 2B** **and** **Fig. 2C**), suggesting that other functions of the protein contributing to virus production, apart from IC activity, may be affected.

### SARS-CoV E protein IC activity improves viral fitness

**Figure 3:**
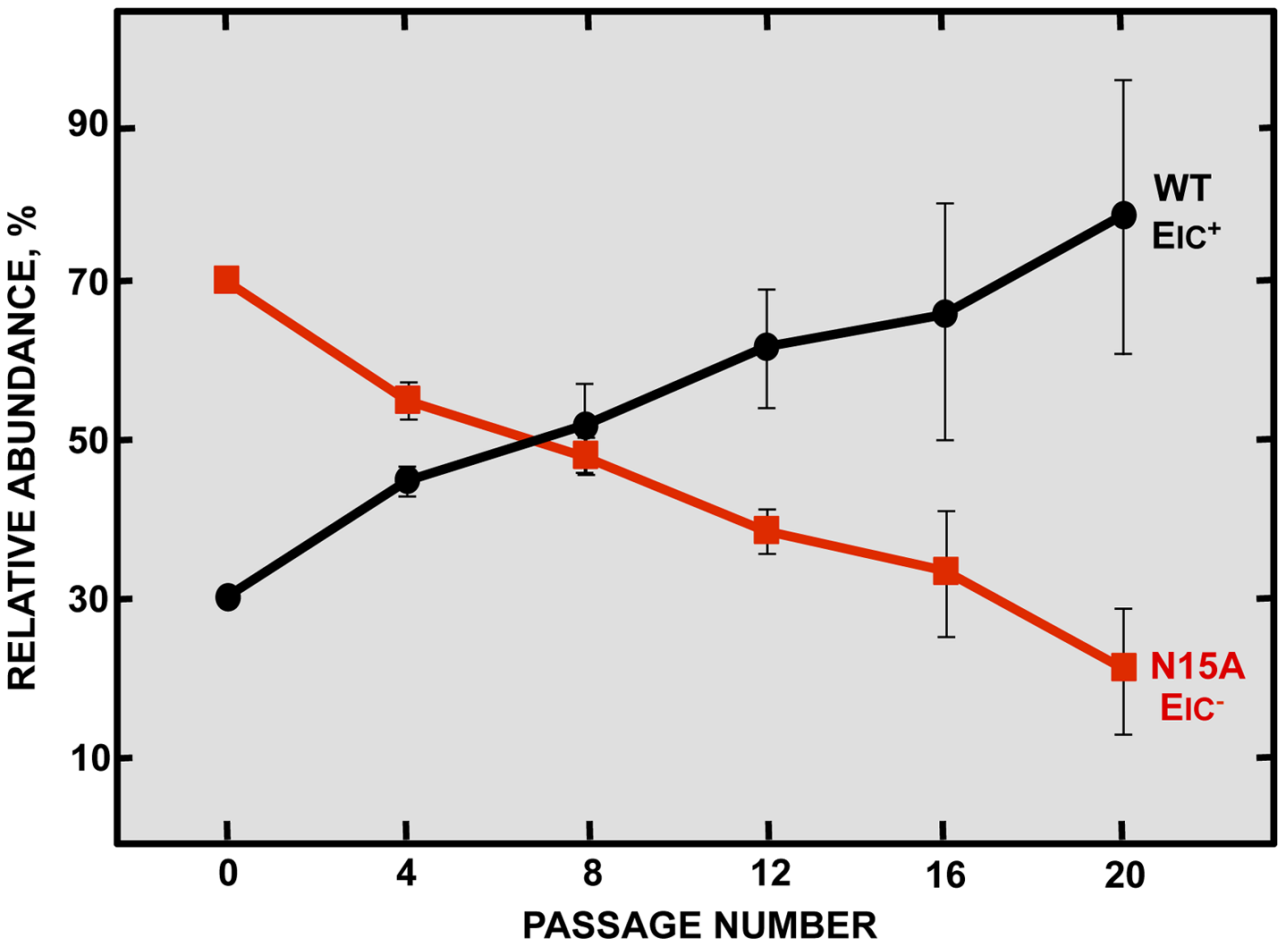
Effect of SARS-CoV E protein IC activity on viral fitness. Competition assays between the parental virus (wt, black circles) displaying IC activity (EIC^+^) and a mutant virus (N15A, red squares) lacking IC activity (EIC^−^) were performed. Vero E6 cells were co-infected with mutant and parental viruses at a ratio 7∶3 and supernatants were serially passaged 20 times every 24 hours. Relative abundance of each virus was determined by sequencing E gene within viral progeny. Error bars represent the standard deviation from three independent experiments.

Inhibition of E protein IC activity slightly reduced virus production in cell culture in a relatively short period of time, but these differences were not significant. To further explore whether ion conductivity could improve viral growth and fitness, a long-term competition assay was performed between the wt virus and the N15A mutant lacking IC activity, that was relative stable through passages as will be described below. Vero E6 cells were co-infected with N15A mutant and the wt virus in a proportion 7∶3, and the supernatant was serially passaged for 20 times every 24 hours. The E gene was sequenced every 4 passages, revealing that the proportion of wt virus steadily increased over the passages, accompanied by a decrease in the abundance of the N15A mutant. From passage 8 on, the wt virus took and maintained majority over the N15A mutant (**Fig. 3**). These results suggested that E protein IC activity for SARS-CoV confers a selective advantage improving virus production.

### SARS-CoV E protein IC activity confers virulence *in vivo*

**Figure 4:**
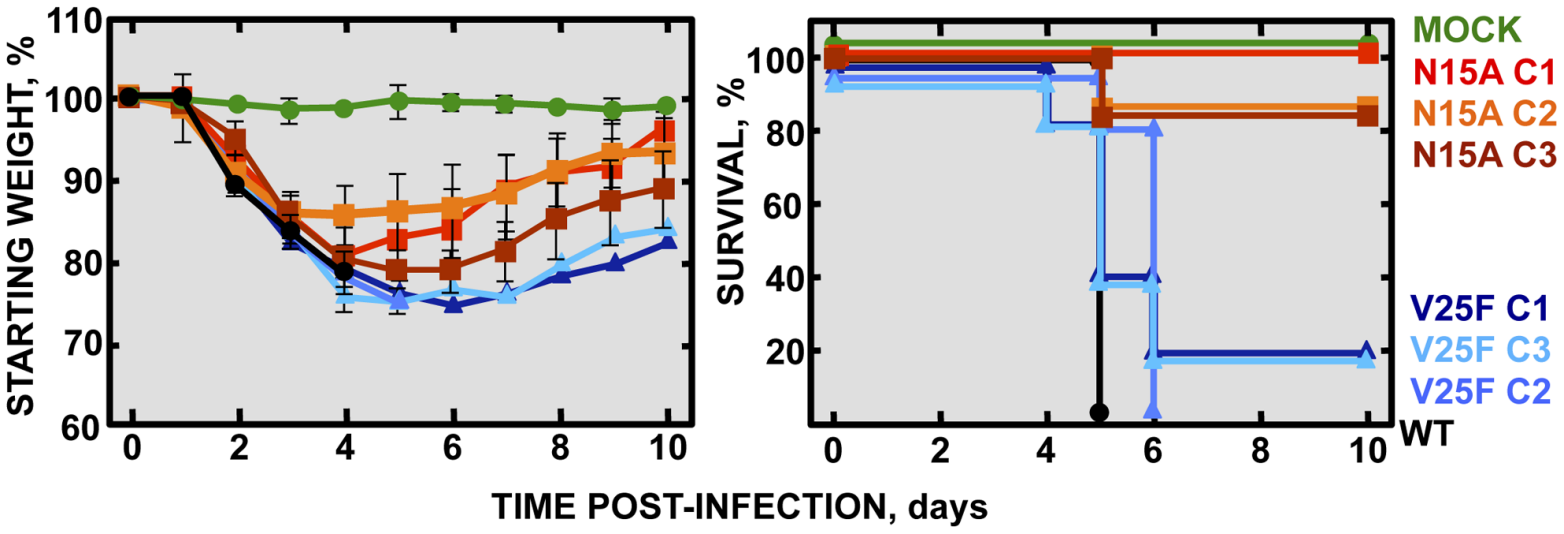
Pathogenesis caused by rSARS-CoV-EIC^−^ in BALB/c mice. Groups of five 16 week-old BALB/c mice were mock infected (Mock, green circles) or infected with 100000 PFU of either the parental virus (wt, black circles) or several clones of the mutant viruses missing IC activity: N15A C1, N15A C2 and N15A C3 (red, orange and deep-red squares, respectively), and V25F C1, V25F C2 and V25F C3 (dark blue, blue and light blue triangles, respectively). Mean weight losses (left graph) and survival (right graph) during 10 days following infection are represented for each group. Error bars represent the standard deviation for mice weights per experimental condition.

**Figure 5:**
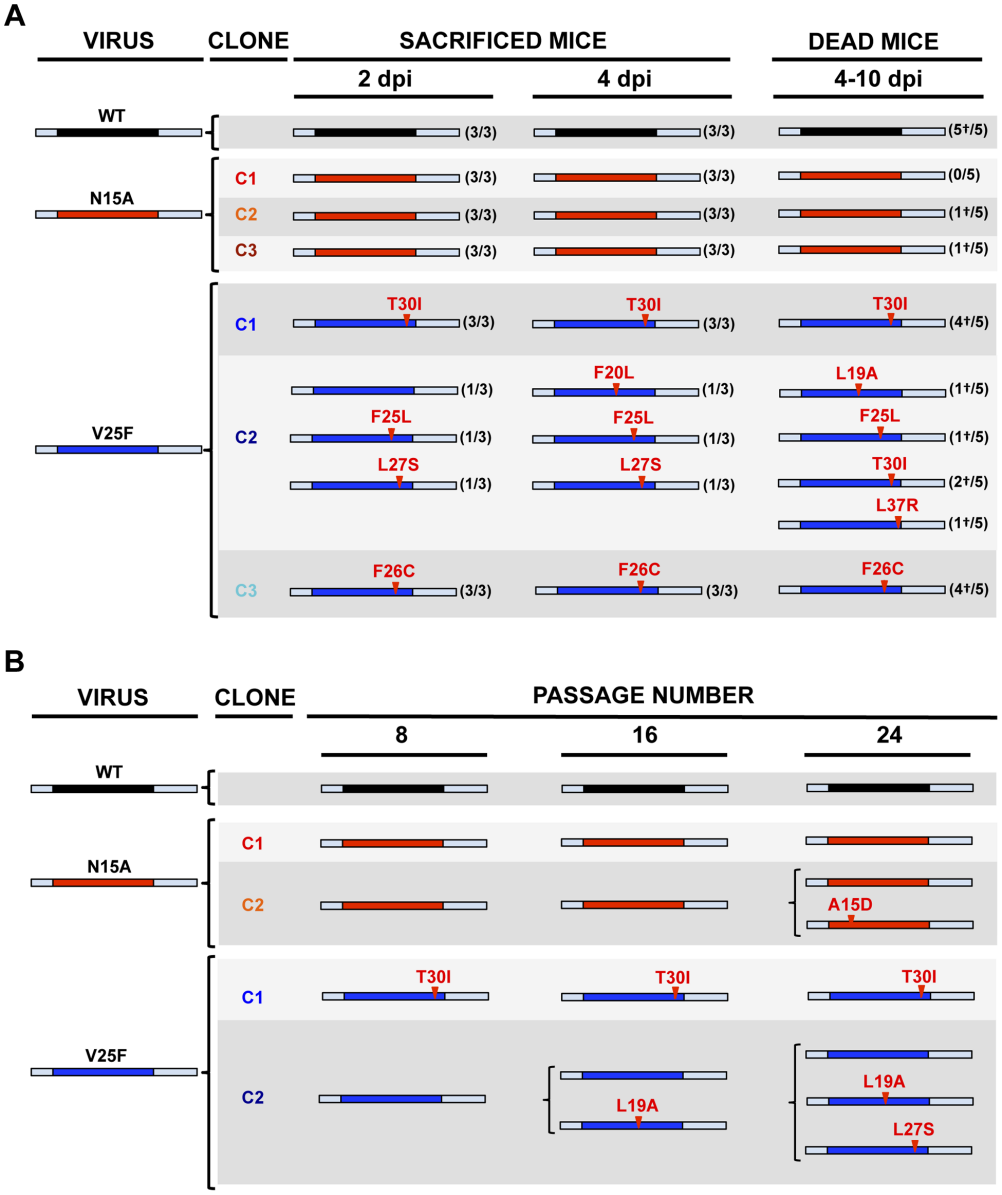
Stability of rSARS-CoV-EIC^−^ after serial infections. (**A**) Groups of eleven 16 week-old BALB/c mice were infected with 100000 PFU of either the parental virus (wt) or three clones of the mutant viruses missing IC activity: N15A C1, N15A C2, N15A C3, V25F C1, V25F C2 and V25F C3. At 2 dpi and 4 dpi 3 mice of each group were sacrificed, lung RNA was extracted, and E gene was sequenced. The rest of the mice (5 per group) participated in the weight-loss and survival experiment. When any mouse died, from 4 to 10 dpi, lung RNA was extracted and E gene was sequenced. Bars represent different E protein sequences, either that of parental or the mutant viruses. The central colored part represents the transmembrane domain of the protein. Letters and numbers in red represent the amino acid changes detected after viral evolution and their relative position within transmembrane domain, respectively. Numbers accompanying bars indicate from how many mice (first number) out of the total of the animals analyzed (second number) arose the indicated sequence change. Dead mice are indicated by a †. (**B**) Vero E6 cells were infected with the wt virus or the mutant clones N15A C1 and N15A C2, V25F C1 and V25F C2 at an initial MOI of 0.5, and supernatants were serially passaged for 24 times every 24 hours. E gene in the viral population was sequenced at passages 0, 8, 16 and 24. Colored bars represent the transmembrane domain of different E protein sequences and letters and numbers in red represent the amino acid mutations identified and their relative position, respectively.

To specifically analyze the contribution of E protein IC activity to SARS-CoV virulence, BALB/c mice were intranasally inoculated with the wt virus displaying E protein IC activity, or three independently-isolated clones of the mutant viruses N15A and V25F lacking E protein IC activity, and mice were monitored daily for 10 days (n = 5/virus clone). All infected animals showed disease symptoms at 2 days post infection (dpi), reflected by slower movements and ruffled fur (data not shown). Mice infected with the wt virus started to lose weight by day 2, and by day 5 all of them died (**Fig. 4**). Interestingly, although mice infected with the three clones of N15A mutant started to lose weight in a similar fashion, at day 4 almost all of them started to regain weight, recover from the disease, and 80–100% survived (**Fig. 4**). In contrast to N15A, mice infected with V25F virus experimented similar weight losses and survival rates (from 0 to 20%) than the wt virus (**Fig. 4**). A possible explanation for this apparent discrepancy was the reversion of the introduced mutation or the incorporation of compensatory mutations restoring E protein IC activity. To test whether this was the case, total RNA was collected from the lungs of infected mice at 2 and 4 dpi or from the lungs of mice that died after infection. The virus genome region containing E gene was sequenced, as it was the target of the point mutations inhibiting IC activity, and therefore a likely place to incorporate compensatory mutations. E genes from wt virus and N15A mutant virus remained stable during the course of the experiment, since no changes were found in viral RNA extracted either from lungs of several mice at 2 and 4 dpi or from dead mice (**Fig. 5A**). In contrast, V25F viruses incorporated mutations in the E gene that led to amino acid changes either in the same position of the mutation that abolished IC activity (F25C) or in relatively close positions within the E protein transmembrane domain: L19A, F20L, F26L, L27S, T30I and L37R (**Fig. 5A**). These evolved variants of the V25F virus appeared as early as 2 days after mice infection and, in some cases (T30I mutant), completely overgrew the original virus by day 2. The tentative compensatory mutations were also present in the viral population at 4 dpi and in dead mice (**Fig. 5A**). Overall, the data obtained with wt and N15A viruses, which were genetically stable throughout the experiment, suggest that E protein IC activity is required for a virulent phenotype.

### Viruses missing E protein IC activity are prone to evolve and restore ion conductivity

**Figure 6:**
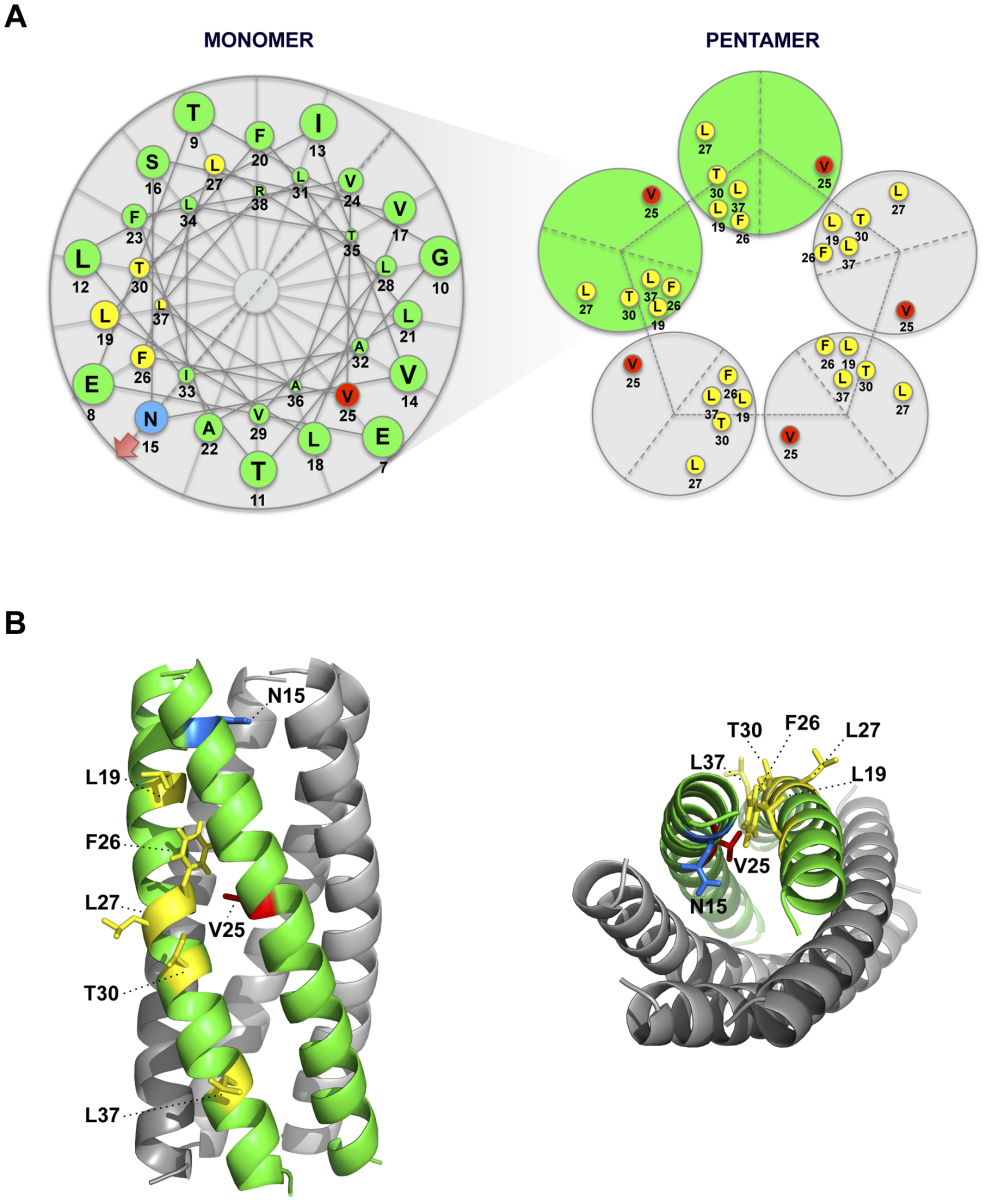
Spatial distribution of the mutations obtained in rSARS-CoV-EIC^−^ after serial infections. (**A**) Left diagram represents a top view of E protein transmembrane domain and the spatial distribution of the amino acids within the alpha helix. Blue and red circles correspond to amino acids N15 and V25, respectively, originally mutated to inhibit IC activity. Yellow circles surround the amino acids that changed after evolution of V25F mutant. Arrow at position 15 points the lumen of the ion channel pore. Right graphic depicts the pentamer conformation of E protein that forms the ion conductive pore and the positions of both the mutated residue at position 25 and the evolved mutations at positions 19, 25, 26, 27, 30 and 37. Evolved changes map close to the originally mutated residue in the monomer-monomer interface. (**B**) Pentameric model of SARS-CoV E protein from a lateral (left) or a top view (right). This model was first proposed from linear dichroism of isotopically labeled E protein transmembrane peptides in lipid bilayers [29], [32]. The residues involved in ion channel inhibition (N15 in blue and V25 in red) or mutated after viral evolution (L19, F26, L27, T30 and L37 in yellow) are highlighted.

**Figure 7:**
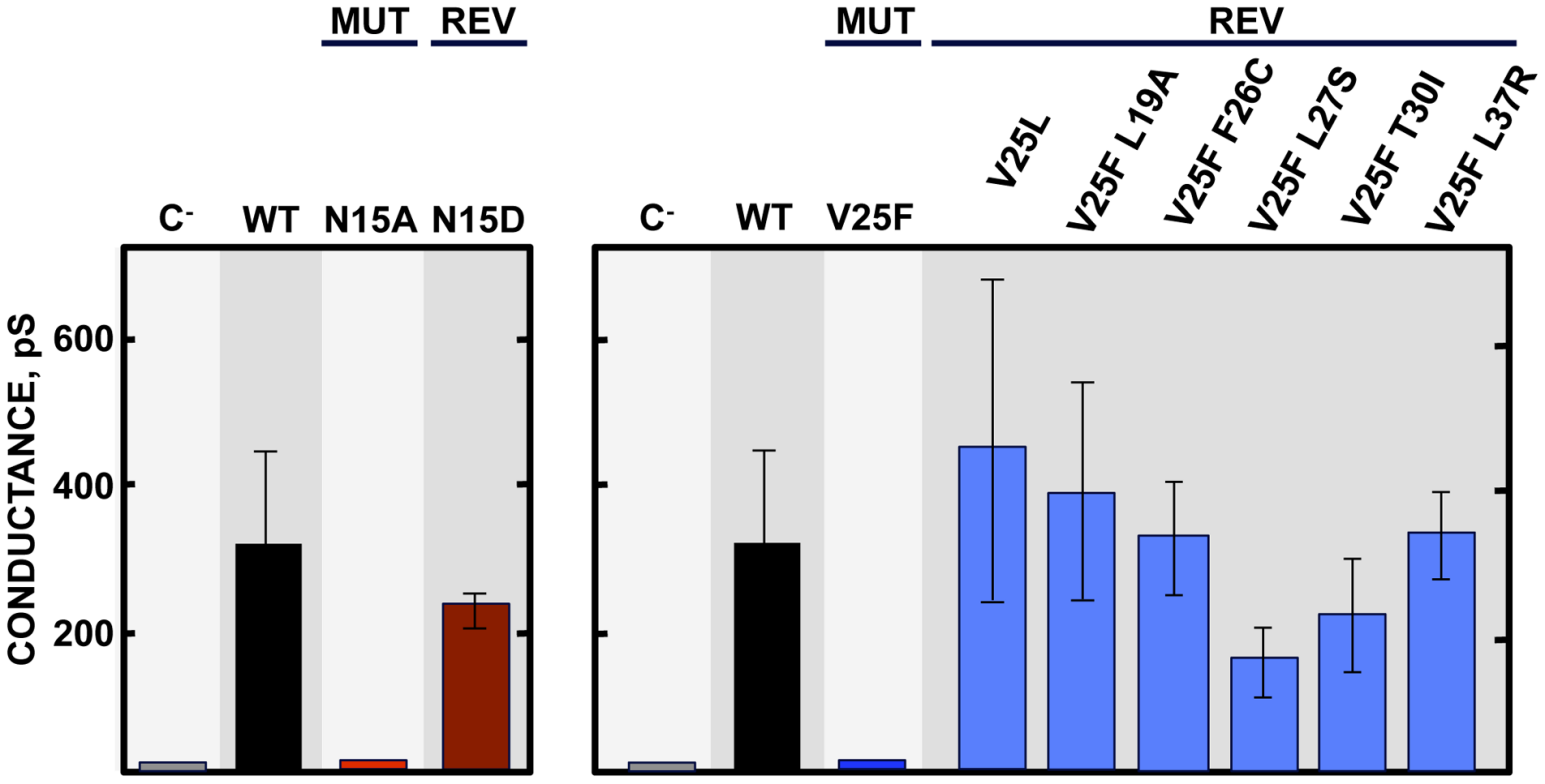
E protein IC activity of the rSARS-CoV-EIC^−^ evolved variants. Synthetic peptides representing E protein transmembrane domain of the parental virus (wt) the mutant viruses (MUT) lacking IC activity (N15A and V25F) and their evolved revertants (REV) obtained after infections of mice or cell culture (N15D, V25L, V25F L19A, V25F F26C, V25F L27S, V25F T30I and V25F L37R) were reconstituted in artificial lipid bilayers, and their IC activity was analyzed as mean conductance values. Negative controls (C^−^) indicate conductance values obtained in the absence of any peptide. Error bars represent the variations obtained in 100 independent experiments.

To further analyze the evolution of the mutant viruses lacking E protein IC activity, two clones of the mutants N15A and V25F were serially passaged in cell culture. Throughout the 24 serial passages, E gene was sequenced at passages 0, 8, 16 and 24 for the two mutant viruses and wt as control. As observed during *in vivo* infection, the wt virus remained stable during the passages (**Fig. 5B**). V25F viruses rapidly incorporated additional mutations within E gene (L19A, L27S and T30I), reproducing our *in vivo* observations. The viruses incorporating T30I mutation completely out-competed the original V25F mutant by passage 8 (**Fig. 5B**). In contrast, N15A viruses either remained stable or incorporated a mutation in the E gene (A15D) that appeared late, at passage 24, suggesting that this mutant was more stable, confirming our *in vivo* results (**Fig. 5B**). The data obtained in cell culture or after mice infection indicate that SARS-CoVs lacking E protein IC activity incorporated mutations at the E gene that directly reverted the original mutation that suppressed IC activity (A15D and F25C) or modified residues mapping to a close position of the E protein transmembrane domain. These modified residues face the original mutation inhibiting IC activity, when the ion channel is assembled (**Fig. 6**). To analyze whether these mutations restored IC activity, synthetic peptides representing the E protein transmembrane domain containing the mutations obtained after viral evolution *in vivo* and in cell culture (N15D, V25L, V25F L19A, V25F F26C, V25F L27S, V25F T30I, V25F L37R), were synthesized. The IC activity of these peptides was evaluated in artificial lipid membranes as previously described [34]. Whereas peptides containing the original mutations N15A and V25F did not show any conductance, all the peptides containing the mutations obtained after viral evolution displayed similar conductance values than a wild type peptide (**Fig. 7**), indicating that all these compensatory mutations restored E protein IC activity.

### Genetically engineered revertant viruses restoring E protein IC activity show a virulent phenotype in mice

**Figure 8:**
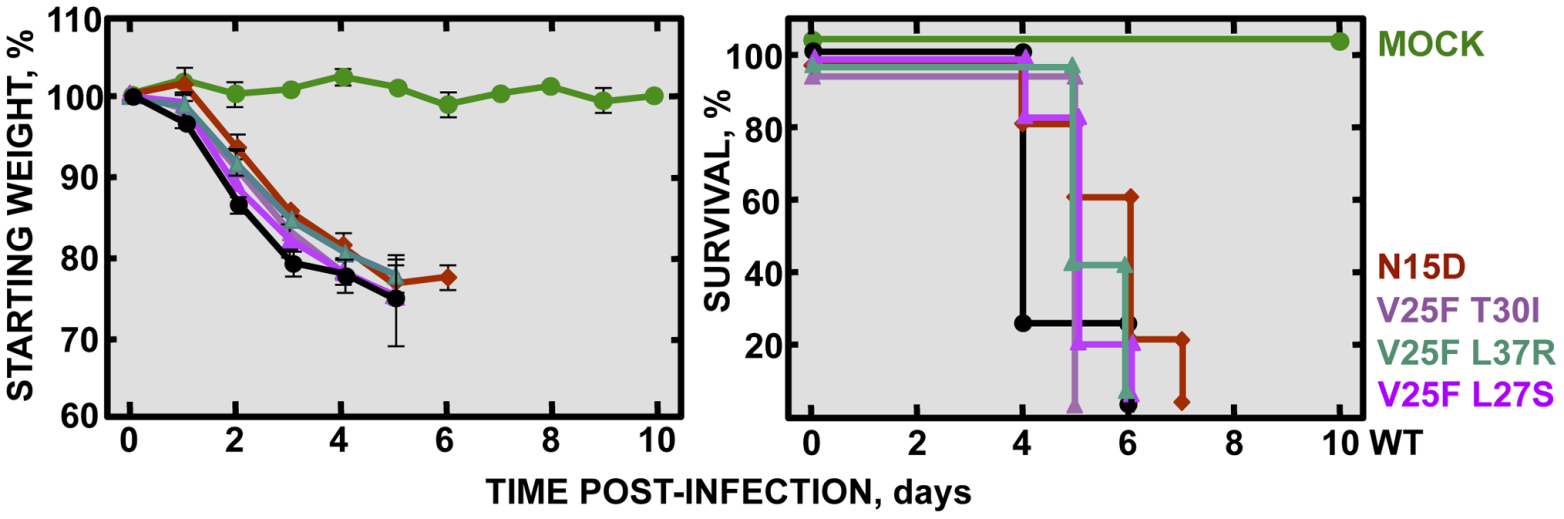
Pathogenesis caused by rSARS-CoV-EIC^rev^ in BALB/c mice. Groups of five 16 week-old BALB/c mice were mock infected (Mock, green circles) or infected with 100000 PFU of either the parental virus (wt, black circles) or the genetically engineered revertant viruses recovering IC activity: N15D (deep-red diamonds), V25F L27S (fuchsia triangles), V25F T30I (pink triangles) and V25F L37R (green triangles). Mean weight losses (left graph) and survival (right graph) during 10 days are represented for each group. Error bars represent the standard deviation for mice weights per experimental condition.

A correlation between IC activity and virulence was found *in vivo*, where N15A viruses lacking IC activity were attenuated compared to wt virus competent in IC activity. Mutant virus V25F, originally lacking ion conductivity, rapidly incorporated compensatory mutations upon infection *in vivo* that restored IC activity and thus caused pathogenicity. To test whether the recovery of IC activity was the unique determinant of virulence, and to rule out effects of other mutations arising outside of the E gene, recombinant viruses containing a set of the compensatory mutations that restored IC activity (rSARS-CoV-EIC^rev^): rSARS-CoV-E-V25F L27S (V25F L27S), rSARS-CoV-E-V25F T30I (V25F T30I), rSARS-CoV-E-V25F L37R (V25F L37R) were engineered, rescued and tested in mice. These viruses were virulent in mice in terms of weight loss and survival rates, causing similar disease as that caused by the wt virus (**Fig. 8**). We sought to confirm this data on another genetic background, so a recombinant SARS-CoV containing the mutation that restored IC activity in N15A mutant after cell culture passage was engineered rSARS-CoV-E-N15D (N15D) and evaluated. In agreement with the V25F revertants, the mutant N15D induced similar morbidity and mortality as wt (**Fig. 8**), confirming that E protein IC activity is a determinant of virus pathogenesis.

### SARS-CoV E protein IC activity is dispensable for efficient growth *in vivo*

**Figure 9:**
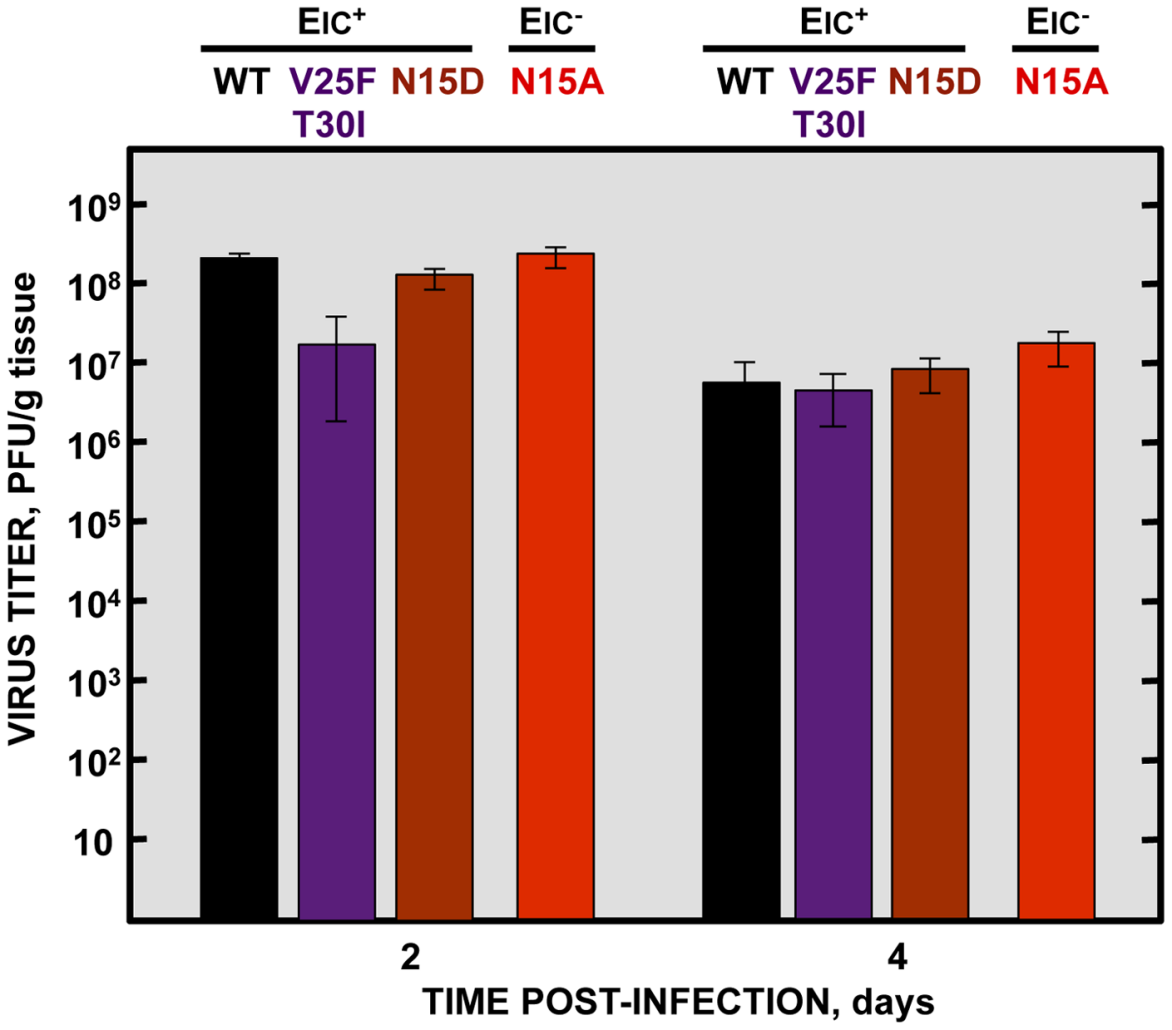
Effects of SARS-CoV E protein IC activity on virus growth in BALB/c mice lungs. Groups of six 16 week-old BALB/c mice were infected with 100000 PFU of viruses displaying E protein IC activity (EIC^+^), either the parental virus (wt, black columns) or the genetically engineered revertant viruses V25F T30I (purple columns) and N15D (deep-red columns) or with the mutant lacking IC activity (EIC^−^) N15A (red columns). At 2 and 4 days post infection (dpi) 3 mice from each group were sacrificed to determine virus titers.

Although E protein IC activity is not essential for virus growth in cell culture (**Fig. 2B**), it is possible that production of virus *in vivo* further depends on ion conductivity. To test if the attenuation observed *in vivo* with IC inactive viruses is due to lower virus production, 16 week-old BALB/c mice were intranasally inoculated with the wt virus, the genetically engineered revertant viruses N15D and V25F T30I displaying IC activity, or the N15A mutant lacking IC activity. Mice lungs were collected at 2 and 4 days post infection, homogenized, and viral titers were determined. Interestingly, the virus lacking IC activity (N15A) grew to the same extent or even better than the wt and the revertant viruses, respectively, reaching titers higher than 10^8^ and 10^7^ PFU/gr of lung tissue at 2 and 4 dpi, respectively (**Fig. 9**). These data indicate that E protein IC activity does not significantly affect virus production *in vivo*, under these experimental conditions. Therefore the attenuation of the virus lacking IC activity is likely due to a host-specific effect mediated by the ion channel in the mouse, and not to a reduction in virus yields.

### Viruses with E protein IC activity induced edema accumulation after SARS-CoV infection

**Figure 10:**
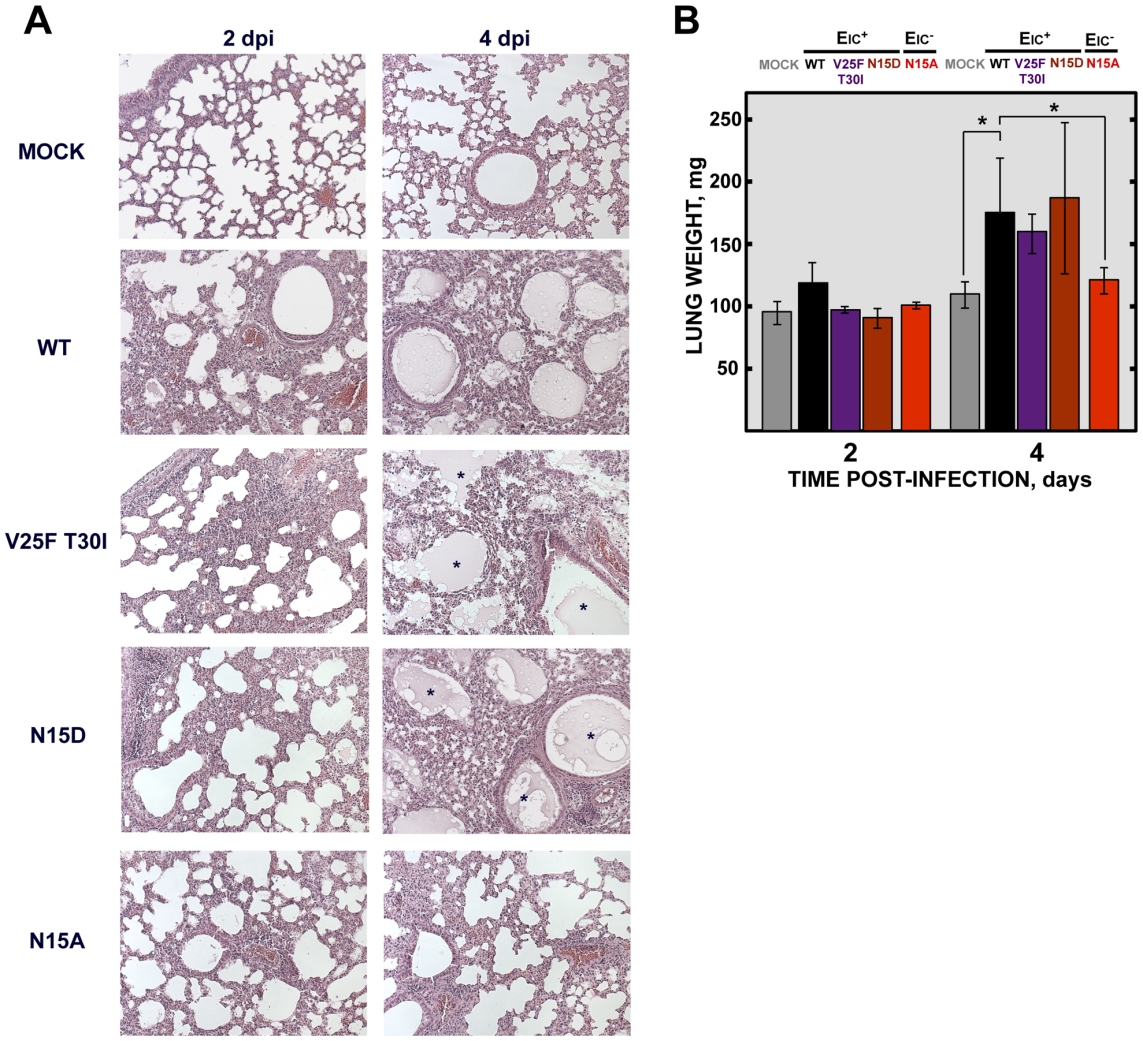
SARS-CoV E protein IC activity and lung pathology. Groups of six 16 week-old BALB/c mice were mock infected (Mock) or infected with 100000 PFU of viruses displaying E protein IC activity (EIC^+^), either the parental virus (wt) or the genetically engineered revertant viruses V25F T30I and N15D or with the mutant lacking IC activity (EIC^−^) N15A. At 2 and 4 dpi 3 mice from each group were sacrificed and their lungs were collected. (**A**) Lungs were fixed in formalin, paraffin embedded, sectioned and processed for hematoxylin and eosin staining. Asterisks indicate edema accumulation in both bronchiolar and alveolar airways. Original magnification was 20×. (**B**) When collected and prior to fixation lungs were weighted. Error bars indicate the standard deviation from 3 mice lungs per each condition. Statistically significant data are indicated with an asterisk (Student's t-test p-value<0.05).

To analyze the mechanisms by which IC inactivity confers less virulence, lung sections of mock-infected mice, or of those infected with the wt virus, IC revertants and N15A mutant were collected at 2 and 4 dpi, stained with hematoxylin and eosin and examined for histopathological changes. Mock-infected animals showed wide free alveolar and bronchiolar airways and no evidence of leukocyte infiltrates (**Fig. 10A**). Animals infected with the viruses displaying IC activity, presented swollen alveoli walls and leukocyte infiltrates in the infected areas at both time points (**Fig. 10A**). The histopathology caused by IC proficient viruses was even more dramatic at 4 dpi, where cell infiltrates were more abundant, and air spaces were collapsed by a profuse lung edema, which is the ultimate cause of acute respiratory distress syndrome (ARDS) that leads to lung failure and death (**Fig. 10A**). Edema accumulation at 4 dpi was also reflected by a marked increase (>1.5 fold) in the weight of lungs in animals infected with viruses competent in E protein ion conductivity (**Fig. 10B**). In contrast, mice infected with the virus lacking IC activity (N15A) showed moderate swollen lung epithelia and lung infiltrates that reflected a productive viral infection. However, at 4dpi, lung airways remained free from pulmonary edema, reflected by both the lung sections and in the minimal change of lung weight (**Fig. 10A and 10B**). Such moderate changes in the lung may retain efficient oxygen exchange. These data suggested that E protein IC activity contributes to SARS-CoV induced lung edema.

### SARS-CoV displaying E protein IC activity induces disassembly of bronchoalveolar epithelia

**Figure 11:**
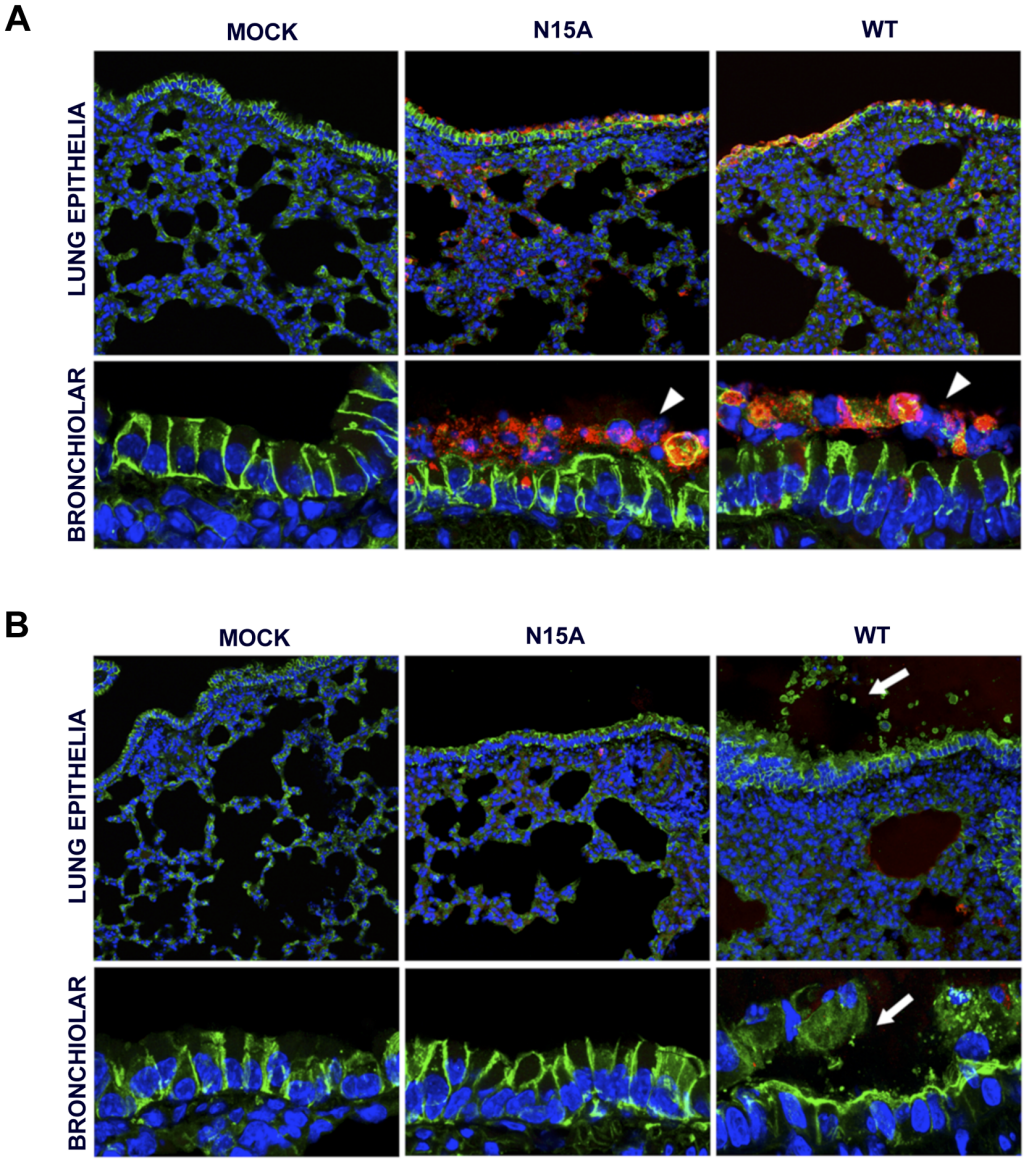
Lung epithelia disassembly in SARS-CoV infected BALB/c mice. 16 week-old BALB/c mice were mock infected (Mock) or infected with 100000 PFU of the parental virus (wt) displaying E protein IC activity or the mutant virus lacking IC activity N15A. At 2 (**A**) and 4 (**B**) dpi mice were sacrificed and their lungs were fixed in formalin, paraffin embedded, sectioned and processed for immunofluorescence. Na^+^/K^+^ ATPase was labeled in green, SARS-CoV N protein was labeled in red to detect infected cells and cell nuclei are shown in blue. A general view of lung epithelia at an original magnification of 40× is shown in the upper rows of the panels. Magnified bronchiolar epithelia at an original magnification of 189× are shown in the rows of the bottom. White arrowheads indicate cell desquamation in the bronchiolar barrier. White arrows show epithelium disassembly and mislocated Na^+^/K^+^ ATPase staining away from basolateral cell membranes, and present within air spaces.

ARDS caused by SARS-CoV infection originates from the accumulation of a protein rich edema, leading to severe hypoxemia and eventually to death. Lung epithelial cells create an osmotic gradient between airways and lung interstitium controlling water levels within air spaces. Damage to the epithelium is therefore a major cause of edema accumulation. To test the correlation between presence of E protein IC activity and an increase in epithelial damage leading to edema accumulation, lungs from mock-infected and from mice infected with the wt or the N15A virus were processed at 2 and 4 dpi for immunofluorescence. Epithelium integrity was evaluated using a specific antibody for Na^+^/K^+^ ATPase, a key factor in establishing the osmotic gradient necessary for edema clearance, and infection was tracked using an antibody specific for N protein. At 2 dpi many infected cells (around 16%) were observed in lungs of mice infected with either wt or N15A virus (**Fig. 11A** **and S1**), overlapping with the most productive time of viral infection. Both viruses presented similar cell tropisms within lungs, infecting bronchiolar epithelium (between 60–70% of the cells) and alveolar epithelium (around 10% of the cells) (**Fig. 11A** **and S1**). Viral infections caused cell death leading to desquamation, especially at the bronchiolar barrier (**Fig. 11A**). At 4 dpi the number of infected cells was dramatically reduced (close to 1%) (**Fig. 11B** **and S1**), accompanying viral titer decrease. Interestingly, wt infected mice showed abundant epithelia disassembly at this time point, especially in the bronchioles. Na^+^/K^+^ ATPase was mislocated from its basolateral position within the plasma membrane of epithelial cells as a consequence of bronchiolar barrier destruction, and detected in desquamated cells or cell debris present at air spaces (**Fig. 11B**), where edema accumulation was also observed (**Fig. 10**). The removal of Na^+^/K^+^ ATPase from its native position within the epithelial barrier most likely prevented its function in edema clearance. In contrast, animals infected with N15A mutant, presented less damaged epithelia and Na^+^/K^+^ ATPase location was not disturbed (**Fig. 11B**), which may allow edema resolution, as no accumulation of protein rich edema was observed under these conditions (**Fig. 10**).

### E protein IC activity triggers the production of IL-1β, TNF and IL-6, key inflammatory cytokines in lung damage and edema accumulation

**Figure 12:**
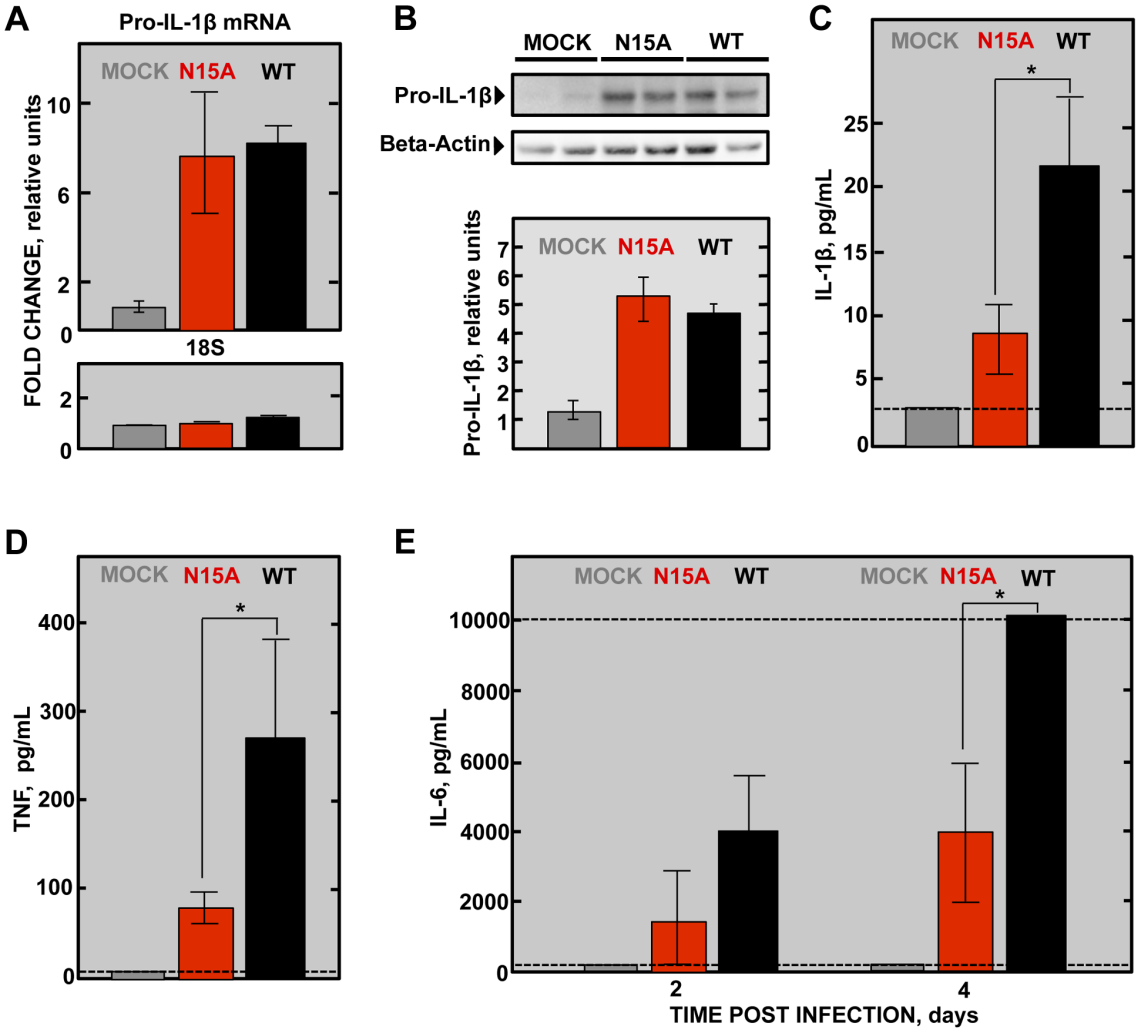
SARS-CoV E protein IC activity and induction of inflammatory cytokines involved in lung injury. Groups of six 16 week-old BALB/c mice were mock infected or infected with 100000 PFU of the parental virus (wt) displaying E protein IC activity or the mutant virus lacking IC activity N15A. At 2 dpi 3 mice from each group were sacrificed and their lungs were collected. (**A**) Total RNA was extracted and levels of pro-IL-1β mRNA, and 18s rRNA (18S), as a control, were analyzed by RT-qPCR. Error bars indicate the standard deviation from samples of 3 mice per experimental setting. (**B**) Lung protein extracts were prepared and levels of inactive pro-IL-1β (35 kDa) and beta-actin, as a loading control, were detected by Western blot and quantified by densitometry analysis. Bottom of the figure represents the ratio pro-IL-1β/beta-actin relative to the mock-infected animals levels, as a reference. Bronchoalveolar lavages (BAL) of infected mice were collected and the concentration of (**C**) the active form of IL-1β protein at 2 dpi, (**D**) TNF protein at 2 dpi and (**E**) IL-6 protein at 2 and 4 dpi within the lavages were determined using the Luminex technology. Error bars indicate the standard deviation from samples of 3 mice per condition. Discontinuous lines indicate the limit of the detection of the technique. Statistically significant data are indicated with an asterisk (Student's t-test p-value<0.05).

Severe damage caused to the epithelial barrier is associated with an acute inflammatory response in the lung parenchyma along with edema accumulation. Elevated levels of inflammatory cytokines IL-1β, TNF and IL-6 are found in the lungs of ARDS patients and play a key role in the progression of the disease [62]. IL-1β is an early response highly inflammatory cytokine that is tightly regulated. During viral infection, recognition of pathogen molecular associated patterns (PAMPs) by the cells, such as double stranded viral RNA, induces IL-1β mRNA expression and translation to generate the inactive form of the protein pro-IL-1β. Upon certain stimuli, pro-IL-1β is then cleaved by caspase-1 through inflammasome activation, generating the active form IL-1β, which is subsequently secreted to exert its function [52]. Interestingly, viral proteins with IC activity have been recently found to activate the inflammasome, which finally leads to the secretion of active IL-1β to the extracellular media [52], [59]. We thus sought to test whether E protein IC activity was implicated in the production of active IL-1β in the lungs of SARS-CoV infected mice. First, the expression of pro-IL-1β mRNA and the amounts of its derived protein, inactive pro-IL-1β, were measured in wt- and N15A-infected mice at 2dpi. Infections with both wt and N15A mutant viruses induced similar overexpression of pro-IL-1β mRNA as compared with the mock-infected animals (**Fig. 12A**). The increased levels of pro-IL-1β mRNA found in infected mice, correlated with enhanced amounts of inactive pro-IL-1β, which reached similar values in wt and N15A infections (**Fig. 12B**). To analyze the levels of active, secreted IL-1β, bronchoalveolar lavages were performed at 2 dpi. The amount of IL-1β in the airways was significantly higher in the mice infected with the wt virus displaying E protein IC activity, over those infected with the mutant N15A missing this function (**Fig. 12C**). Collectively, these data indicated that E protein IC activity promotes the secretion of mature IL-1β, without increasing pro-IL-1β transcription or synthesis.

IL-1β enhances the production of TNF, another key early response cytokine, and IL-6, which follows a more sustained increase over time [62]–[64]. Therefore, it is not surprising that both TNF and IL-6 levels were more dramatically increased in wt-infected mice in comparison with the N15A-infected mice at 2 dpi (**Fig. 12D and 12E**). Furthermore, analysis of IL-6 levels in the bronchoalveolar lavages of infected mice at 4 dpi, revealed that overwhelming amounts of this cytokine, exceeding 10000 pg/mL, accumulated in wt-infected mice, whereas IL-6 levels were at least 2.5-fold lower when E protein IC activity was absent during infection (**Fig. 12E**). All these results indicate that the presence of E protein IC activity correlates with the activation of the inflammasome and an acute inflammatory response that is deleterious for lung tissue.

## Discussion

Several viruses that cause severe diseases in humans encode small transmembrane proteins containing IC activity [44]. The alteration of host cell ion balance by these proteins is usually necessary for virus production and maturation, but the effect of IC activity in pathogenesis is less well understood. Coronaviruses are the causative agent of recent and likely future serious diseases. We have focused this study on SARS-CoV E protein, a virulence determinant displaying IC activity. In this manuscript, we sought to elucidate the role of E protein IC activity in virus pathogenesis by combining our knowledge of residues essential for E protein ion conductivity with the manipulation of SARS-CoV genome. To this end we used a mouse adapted genetic background (MA15) assembled in a bacterial artificial chromosome (BAC). Two rSARS-CoVs, each one containing mutation N15A or V25F in the transmembrane domain of E protein were generated to knock down its IC activity. Upon competition during several passages, the viruses lacking E protein IC activity were clearly overgrown by the parental virus, which replicated better. Nevertheless, these differences in viral growth needed several replication cycles to be amplified and detected, as only slight no significant changes in virus production were observed after 72 hours growth kinetics. In agreement with this result, when T16A mutation was introduced within IBV E protein, which represents the equivalent mutation to SARS-CoV E protein N15A, no alterations in the production of virus like particles (VLPs) were detected after 48 hours [40]. The fact that deeper alterations of CoV E protein transmembrane domain cause much more dramatic effects in virus production [37], [39] may be due to additional structural or functional changes in E protein, besides their effect on ion conductivity. In conclusion, E protein IC activity, although not essential for virus production, confers and advantage to the virus by enhancing its fitness and growth. Accordingly, a selective advantage of IC activity has also been shown for influenza virus. Mutants lacking M2 protein IC activity were overgrown by the parental virus in competition assays in an even faster manner than in SARS-CoV, probably because the influenza virus lacking IC activity has more profound replication defects [65], [66]. SARS-CoV encodes other two proteins, 3a and 8a, which also contain IC properties [41], [42]. Therefore, an essential contribution of IC activity to virus production cannot formally be excluded for SARS-CoV, as 3a and 8a derived ion channels could functionally compensate the absence of E protein IC activity.

SARS-CoV mutant viruses lacking E protein IC activity showed a clear tendency to revert both in cell culture and *in vivo* after mice infection. N15A and V25F mutant viruses, devoid of E protein ion conductivity, incorporated additional mutations in the E gene to restore IC activity, suggesting that this function confers a selective advantage to the virus. This trend was more evident in the case of V25F virus, which evolved more quickly and frequently than the N15A mutant virus. No reversion of E protein IC activity was observed for N15A mutant in mice, at least during the first five days post infection. Attempts to sequence the viral progeny at 9 and 10 days after the inoculation were unsuccessful, probably because the virus was mostly cleared by those time points. Nevertheless, as N15A mutant restored its ion channel activity after long number (>24) of passages in cell culture, it is possible that after serial passages *in vivo* this mutant could also revert, as ion conductivity confers better fitness for the virus. Although both N15A and V25F mutations equally disrupted IC activity, the mechanisms by which this is achieved could be different. Replacement of N at position 15 to A, an amino acid predicted to be located facing the channel lumen, is not likely to affect the channel architecture. In fact, the rotational orientation in lipid bilayers of a labeled synthetic transmembrane peptide bearing this mutation was entirely consistent with that of a pentameric model [29]. In contrast, mutation at V25 implies the introduction of a larger side chain (replacement of V to F) at the monomer-monomer interface, which is likely to affect the overall structure of the homo-oligomer and therefore inhibit ion conductivity by causing larger structural changes. This may also explain the higher number of compensatory mutations found in V25F with respect to N15A virus, as the ways to recover a stable oligomer are more varied than those needed to recover channel activity. The compensatory mutations incorporated by the V25F mutant mapped to the opposite face of the transmembrane helix, although they are adjacent when the E protein pentamer is formed. Therefore, the compensatory mutations most likely restored the interaction and assembly between the mutated monomers, reinforcing our hypothesis (**Fig. 6**). IC activity restoration through virus passage suggests that this function is important for the virus. Several of the mutations restoring ion channel activity appeared both in mice and in cell culture. Therefore, it seems that reverting E protein ion conductivity, and not adaptation to mice, was its main goal. Nevertheless, the possibility that these mutations could also improve mouse adaptation through an ion channel dependent or independent mechanism cannot be fully excluded.

E protein IC activity was also involved in SARS-CoV pathogenesis as tested in the mouse model. Viruses lacking IC activity that were stable during multiple passages (N15A mutants) caused reduced mortality, whereas the wt and the mutant viruses restoring IC activity during mice infection (V25F background-evolved variants) caused high mortality rates. Furthermore, genetically engineered viruses containing the point mutations necessary to recover E protein IC activity induced similar mortality as wt virus, reinforcing that E protein IC activity contributes to SARS-CoV pathogenicity. The relevance of viroporins in virus virulence has also been shown in other viruses, such as respiratory syncytial virus SH protein, influenza A virus M2 protein and classical swine fever virus p7 protein [67]–[70], by deleting a large fraction of or the entire protein. Viroporins may play other critical functions apart from ion conduction. Therefore, a direct correlation between IC activity and virulence could not be formally established. To our knowledge, this is the first time in which the IC activity of a viroporin is directly linked to the virulence of the virus.

The infection with highly pathogenic respiratory viruses, including SARS-CoV, is one of the causative agents of acute lung injury (ALI) and its most severe form, ARDS [71]. Just in the United States, 200,000 ARDS cases are reported annually with a 40% mortality rate [72]. Late stages of ARDS are characterized by development of pulmonary edema that leads to an impaired gas exchange, hypoxemia and eventually death. Infection of mice with rSARS-CoV-MA15 resulted in an abundant edema accumulation both in alveolar and bronchiolar spaces at late times post infection (4 dpi), which correlated with mortality. This phenotype was reproduced upon mice infection with other highly-virulent SARS-CoVs displaying IC activity based on alternative E protein sequences (revertant viruses). On the other side, infection of mice with the attenuated mutant lacking E protein IC activity (N15A) caused significantly reduced edema accumulation, likely contributing to a majority of the animals surviving. Collectively, these data indicate that E protein IC activity *in vivo* promotes lung pathology through edema accumulation.

The pulmonary epithelia regulate water levels present within air spaces, a critical parameter for gas exchange, and play a critical role in edema clearance [72], [73]. Epithelial cells create an osmotic gradient mainly through a coordinated Na^+^ transport first from the airways to the cell cytoplasm through epithelial sodium channels (ENaC), located at the apical part of the plasma membrane, and then to the interstitium by Na^+^/K^+^ ATPase, present at the basolateral region of the plasma membrane. This vectorial transport of Na^+^ is accompanied by a water removal from the airspace and edema resolution [72], [73]. The integrity of alveolar and bronchiolar epithelia was analyzed by labeling of Na^+^/K^+^ ATPase in the lungs of mice infected with the virus containing or lacking IC activity. Interestingly, animals infected with the wt virus presented a strong disassembly of bronchiolar epithelia and mislocalization of Na^+^/K^+^ ATPase from its basolateral distribution within cells at late times, coincident with edema accumulation. In contrast, epithelia integrity was clearly preserved in the lungs of animals infected with the virus missing E protein IC activity. Intact lung epithelia may be required for proper function of the main components involved in edema resolution (Na^+^/K^+^ ATPase and ENaC), which may explain the lack of edema and therefore the attenuation observed for this virus. As previously described for SARS-CoV, differences in viral tropism within lung cells, without affecting viral production, can induce different pathologies [74]. Nevertheless, we have observed no significant differences in the infection patterns in the presence or absence of E protein ion channel activity, suggesting that the virulence conferred by E protein IC activity does not depend on alternative tropisms.

Pulmonary epithelia damage leading to ALI and ARDS is a consequence of a cytokine burst initiated, in this case, by viral infection. One of the key early-response cytokines driving proinflammatory activity in bronchoalveolar spaces is IL-1β [75]. IL-1β is mainly produced by macrophages and dendritic cells through inflammasome activation. Ion imbalances within cells have been described as triggers of this pathway [52]. The levels of active IL-1β secreted to the airways were enhanced when E protein IC activity was conserved in SARS-CoV infection. Taking into account that the presence or absence of SARS-CoV E protein IC activity did not interfere with the production of IL-1β precursors (mRNA and protein levels of pro-IL-1β), these results suggest that E protein ion conduction may induce inflammasome triggering resulting in secretion of mature IL-1β. In agreement with this hypothesis, release of active IL-1β has recently been reported for viroporins of other viruses [52], [53], [57], [59]. IL-1β is implicated in the development of diverse pathologies, including obesity, atherosclerosis, diabetes and several pulmonary illnesses such as asthma, pulmonary obstructive chronic disease and ARDS progression through edema accumulation [75]–[78]. Here, we report the implications of this cytokine in SARS-CoV pathology and E protein ion channel activity as a trigger of its production.

ARDS progression involves the production of TNF, another early response cytokine, and IL-6, which exerts its function in a more sustained manner accumulating during the disease [63]. We found that after SARS-CoV infection, these patterns of cytokine expression were clearly reproduced. TNF and IL-6 accumulated to higher levels in the lungs of animals infected with the wt virus displaying IC activity compared to the mutant lacking ion conductivity. IL-1β enhances the production of TNF, and IL-6 is stimulated by both cytokines providing an integrated amplified inflammatory response, detrimental for pulmonary function [63]. Elevated amounts of these cytokines have been reported in bronchoalveolar lavages of SARS-CoV patients [79]. Therefore, the enhanced amounts of active IL-1β found in the animals infected with the wt virus may explain the increased levels of TNF and IL-6, which leads to severe pathology. It is important to note that the increased damage found in pulmonary epithelia infected with the virus displaying E protein IC activity may not be explained by a higher virus production, as suppression of E protein IC activity rendered similar growth in mice lungs during the analyzed time points. SARS-CoV early replication may be a relevant issue in the induction of pathology. We cannot exclude early replicative defects for the N15A mutant in mice, delaying virus growth during the first hours post-infection. Nevertheless, alternative explanations are also possible, as it has been described that some mutations at SARS-CoV S gene conferred increased virulence without affecting growth within mice, even at early times. This increased pathogenesis was mainly dependent on an exacerbated host response to the viral infection [74]. Accordingly, the enhanced inflammatory response triggered by E protein ion channel proficient viruses may be a major pathology inducer.

In this study, we have shown that SARS-CoV E protein IC activity is a virulence determinant, influencing inflammatory responses, including those inflammasome-derived, pulmonary damage and disease outcome. Although not essential for virus production, E protein IC activity confers a selective advantage, as the parental virus, competent for ion conductivity, was more fit. Nevertheless, the virulence associated to E protein ion conductivity could represent a non-selectable consequence. SARS-CoV crossed species barriers from zoonotic reservoirs such as bats, palm civets and raccoon dogs to humans, causing a severe disease [71]. Possibly, in SARS-CoV infection of its natural hosts, E protein ion channel activity may not have a relevant impact in SARS-CoV pathogenesis, and therefore it was positively selected before crossing species barrier. In conclusion, this work provides several findings that may have translational relevance for other coronaviruses, such as the highly pathogenic MERS-CoV, and even on other viruses encoding proteins with IC activity.

## Materials and Methods

### Ethics statement

Animal experimental protocols were approved by the Ethical Committee of The Center for Animal Health Research (CISA-INIA) (permit numbers: 2011-009 and 2011-09) in strict accordance with Spanish national Royal Decree (RD 1201/2005) and international EU guidelines 2010/63/UE about protection of animals used for experimentation and other scientific purposes and Spanish national law 32/2007 about animal welfare in their exploitation, transport and sacrifice and also in accordance with the Royal Decree (RD 1201/2005). Infected mice were housed in a ventilated rack (Allentown, NJ).

### Cells

The African green monkey kidney-derived Vero E6 cells were kindly provided by Eric Snijder (Medical Center, University of Leiden, The Netherlands). The mouse delayed brain tumor cells stably expressing the murine receptor for SARS-CoV (DBT-mACE2) were generated as previously described [61]. Baby hamster kidney cells (BHK-21) were obtained from American Type Culture Collection (ATCC; CCL-10). Cells were grown at 37°C with an atmosphere of 98% humidity, in Dulbecco's modified Eagle medium (DMEM, GIBCO) supplemented with 25 mM HEPES, 2 mM L-glutamine (SIGMA), 1% non essential amino acids (SIGMA) and 10% fetal bovine serum (FBS, Biowhittaker).

### Mice

Specific-pathogen-free 8 week-old BALB/c OlaHsd female mice were purchased from Harlan. Mice were maintained for 8 additional weeks in the animal care facility at the National Center of Biotechnology (Madrid). All protocols were approved by the Ethical Review Committee at the center for animal health research (CISA-INIA). For infection experiments, 16-week-old mice females were anesthetized with isoflurane and intranasally inoculated with 100000 PFU of the indicated viruses. All work with infected animals was performed in a BSL3 laboratory (CISA, INIA) equipped with a ventilated rack (Animal transport unit-Bio containment unit, Harvard) to store the animals during the experiment.

### Construction of mutant rSARS-CoVs-MA15

An infectious cDNA clone encoding a mouse adapted (MA15) SARS-CoV assembled in a bacterial artificial chromosome (BAC) in our laboratory [26] was used as the background to introduce the mutations that inhibited or restored E protein IC activity. Briefly, DNA fragments representing the nucleotides 26044 to 26779 of SARS-CoV-MA15 genome, flanked by the restriction sites BamHI and MfeI, respectively, were chemically synthesized (Bio Basic Inc). These fragments contained different point mutations within the E gene, that generated amino acid changes inhibiting IC activity: N15A (AAT to GCC) and V25F (GTA to TTC) or restoring this activity: N15D (GCC to GAC), V25F L19A (GTA to TTC and CTT to GCA), V25F L27S (GTA to TTC and TTG to TCG), V25F T30I (GTA to TTC and ACA to ATA), V25F L37R (GTA to TTC and CTT to CGT). The fragments containing these mutations were digested and exchanged in the original BAC. The genetic integrity of the cloned DNA was verified by restriction analysis and sequencing.

### Recovery of recombinant viruses from the cDNAs clones

BHK cells were grown to 95% confluency in 12.5 cm^2^ flasks and transfected with 6 µg of the infectious cDNA clones and 18 µl of Lipofectamine 2000 (Invitrogen), according to the manufacturer's specifications. At 6 hours post transfection, cells were trypsinized, added to Vero E6 cells confluent monolayers grown in 12.5 cm^2^ flasks and incubated at 37°C for 72 h. Cell supernatants were harvested, passaged once on fresh cells and the recovered viruses were cloned by three rounds of plaque purification following standard procedures.

### Growth kinetics and plaque assays

Vero E6 or DBT-mACE2 cells were grown to confluency on 12.5 cm^2^ flasks and infected at a multiplicity of infection (MOI) of 0.001. Cells supernatants were collected at 0, 6, 24, 48 and 72 hpi and titrated on Vero E6 cells. For virus titration and plaque detection, supernatants of infected cells were added to confluent monolayers of Vero E6 cells and incubated for 45 min at 37°C. Media was removed and cells were overlaid with DMEM containing 0.6% of low melting agarose and 2% of fetal calf serum (FCS). At 72 hpi cells were fixed with 10% formaldehyde and stained with crystal violet.

### Confocal microscopy

Vero E6 cells were grown to 90% confluency on glass coverslips and infected with rSARS-CoV-ΔE, rSARS-CoV wt, rSARS-CoV-E-N15A and rSARS-CoV-E-V25F at an MOI of 0.3. At the indicated hpi, media were removed and cells were washed twice with PBS and fixed with 4% paraformaldehyde in PBS for 30 min at room temperature. Then, cells were washed twice with PBS and permeabilized for 10 min with 0.2% saponin and 10% FBS in PBS. Primary antibody incubations were performed in PBS containing 10% FBS and 0.2% saponin for 1 h 30 min at room temperature. Immunofluorescence was performed using a mouse mAb specific for ERGIC53 (dilution 1∶200, Alexis Biochemicals), and a rabbit pAb specific for E protein [15] at 1∶2000 dilution. Coverslips were washed four times with PBS between primary and secondary antibody incubations. Alexa 488- or Alexa 546-conjugated antibodies specific for the different species (dilution 1∶500, Invitrogen) were incubated for 45 min at room temperature in PBS containing 10% FBS. Nuclei were stained using DAPI (dilution 1∶200, Sigma). Coverslips were mounted in ProLong Gold anti-fade reagent (Invitrogen) and examined on a Leica SP5 confocal microscope (Leica Microsystems). Colocalization studies were performed using Leica LAS AF v2.6.0 software.

### Virus genome sequencing

The genomic region including nucleotides 26017 to 26447 that contains the E gene was sequenced after RT and PCR reactions. Briefly, total RNA from infected cells or homogenized mice lungs, was collected and purified using RNeasy kit (Qiagen) according to the manufacturer's specifications. For RT reaction, 1 µg of RNA was used as template, random oligonucleotides primers and Thermoscript reverse transcriptase (Invitrogen). The product was subsequently subjected to a PCR reaction using the oligonucleotides E-VS (CTCTTCAGGAGTTGCTAATCCAGCAATGG) and E-RS (TCCAGGAGTTGTTTAAGCTCCTCAACGGTA) and the Vent polymerase (New England Biolabs), following manufacturer's recommendations. Sequence assembly and comparison with the consensus sequence of SARS-CoV-MA15 strain were performed with the SeqMan program (Lasergene, Madison, WI).

### Genetic stability through serial infections of SARS-CoVs lacking IC activity

Confluent monolayers of Vero E6 cells grown in 12.5 cm^2^ flasks were infected at an MOI of 0.5 with the viruses rSARS-CoV wt, rSARS-CoV-E-N15A and rSARS-CoV-E-V25F. At 24 hpi, supernatants were collected and passaged on fresh monolayers of Vero E6 cells, performed 24 times, serially. E gene sequence was analyzed at passages 0, 8, 16 and 24 as described.

For *in vivo* experiments, mice were intranasally inoculates with 100000 PFU of the viruses described above. Lungs were collected at days 2 and 4 post infection and incubated in RNAlater (Ambion) at 4°C for 48 hpi prior to −80°C freezing. To extract total RNA, lungs were homogenized in 2 ml of RLT lysis buffer (QIAGEN) containing 1% β-mercaptoethanol using gentleMACS Dissociator (Miltenyi Biotec). Samples were centrifuged at 3000 rpm during 10 min, and RNA was purified from supernatants using RNeasy kit (QIAGEN) as previously described.

### Peptide synthesis and ion channel measurements in artificial lipid membranes

Synthetic peptides representing the transmembrane domain of SARS-CoV E protein (amino acids 7 to 38) encoding the point mutations that appeared after serial infections of the mutant viruses were generated by standard phase synthesis, purified by HPLC and their IC activity was tested in artificial lipid membranes, as previously described [34].

### Competition assays

Total RNA from co-infected cells was isolated and E gene was sequenced as described above. Relative abundance of the rSARS-CoV wt and rSARS-CoV-E-N15A viruses within viral population was determined by quantifying the relative amounts of their respective E gene genetic markers in the sequence obtained from the population.

### Mice infection and evaluation of virus virulence

16 week-old BALB/c mice females were intranasally inoculated with 100000 PFU of the viruses rSARS-CoV wt, rSARS-CoV-E-N15A, rSARS-CoV-E-V25F, rSARS-CoV-E-N15D, rSARS-CoV-E-V25F L27S, rSARS-CoV-E-V25F T30I and rSARS-CoV-E-V25F L37R in 50 µl of DMEM containing 2% FCS. Weight loss and survival of the infected mice were monitored for 10 days. Animals reaching weight losses higher than 25% of the initial body weight were sacrificed according to the established euthanasia protocols.

### Virus growth in mice lungs and lung histology

Mice were inoculated with 100000 PFU of the virus rSARS-CoV wt, rSARS-CoV-E-N15A, rSARS-CoV-E-N15D and rSARS-CoV-E-V25F T30I, sacrificed at days 2 and 4 post infection, and lungs were collected. To analyze viral growth, right lungs were homogenized in 2 ml of Phosphate Buffered Saline (PBS) containing 100 UI/ml penicillin, 100 µg/ml streptomycin, 50 µg/ml gentamicin and 0.5 µg/ml fungizone using MACS homogenizer (Miltenyi Biotec) according to manufacturer's protocols, and titered as previously described. To examine lung histopathology, left lungs of infected mice were incubated with 10% zinc formalin for 24 h at 4°C, embedded in paraffin, sectioned, and stained with hematoxylin and eosin.

### Immunofluorescence in lung sections

Five micron sections of zinc formalin fixed lungs were deparaffined at 60°C and rehydrated by successive incubations in 100% xylol, 100% ethanol and 96% ethanol. Antigen unmask was performed by boiling the samples in citrate buffer (8.2 mM sodium citrate; 1.8 mM citric acid, pH 6.5) for 5 min at 110°C in a decloaking chamber (Biocare medical). Samples were then permeabilized with 0.25% Triton X-100 in PBS for 15 min and blocked with 10% bovine serum albumin (BSA) and 0.25% Triton X-100 in PBS for 30 min. Samples were labeled with a mouse monoclonal antibody specific for SARS-CoV N protein (kindly provided by Ying Fang, South Dakota State University) diluted 1∶250 and a rabbit monoclonal antibody specific for Na^+^/K^+^ ATPase alpha subunit (Abcam) diluted 1∶100 in 0.25% Triton X-100 and 10% BSA in PBS for 1 h 30 min at room temperature. Goat anti-mouse and goat anti-rabbit secondary antibodies bound to Alexa 488 and Alexa 594 fluorophores were used respectively at a dilution 1∶250 in 0.25% Triton X-100 and 10% BSA in PBS for 45 min at room temperature. Cell nuclei were stained with DAPI (1∶200). Tissues were mounted in ProLong antifade reagent (Invitrogen) and analyzed in a Leica TCS SP5 confocal microscope.

### RT-qPCR analysis

RNA extracted from lungs of infected mice was prepared as described above, and subjected to retro transcriptase reactions using a High-Capacity cDNA transcription kit (Applied Biosystems) to generate cDNAs. PCR using Taqman assays specific for IL-1β (Mm01336189-m1) and 18S ribosomal RNA as a control (Mm03928990-g1) [80], [81] (Applied Biosystems) were performed. Data were acquired with an ABI Prism 7000 sequence detection system (Applied Biosystems) and analyzed using ABI Prism 7000 SDS v1.0 software. Gene expression relative to mock-infected samples is shown.

### Lung protein extracts preparation and western blot assays

Lungs from infected mice were collected at 2 dpi and the right lung was homogenized in 1.2 mL of protein lysis buffer containing Tris/HCl 10 mM, EDTA 1 mM, NaCl 150 mM, IGEPAL 1%, and complete protease inhibitor (Roche) pH8, using MACS homogenizer (Miltenyi Biotec). Samples were centrifuged for 1 h at 4°C and 13000× g and supernatants were collected. Pro-IL-1β levels were analyzed by Western blotting using a goat anti mouse IL-1β/IL-1F2 antibody (R&D systems). As a loading control, beta-actin was labeled using a mouse monoclonal antibody (Abcam). Bound antibodies were detected using a rabbit anti goat and a rabbit anti mouse HRP conjugated antibodies and the Immobilon Western chemiluminiscence substrate (Millipore), following manufacturer's specifications. Densitometric analysis was performed in non-saturated exposures of several experimental replicates using Quantity One, version 4.5.1 software (BioRad). Levels of pro-IL-1β were normalized to the levels of beta-actin.

### Bronchoalveolar lavages

Following euthanasia by cervical dislocation, the trachea was exposed and cannulated through the animal mouth with a 19 gauge tube. Lungs were lavaged three times with 400 µl of cold phosphate buffered saline (PBS). Samples were centrifuged for 10 minutes at 3000× g at 4°C to separate cellular content, and supernatants were collected to analyze their cytokine levels.

### Cytokine multiplex analysis

Bronchoalveolar lavages were treated with IGEPAL reaching a final concentration of 0.2%, to inactivate sample infectivity. The expression of IL-1β, TNF and IL-6 was measured using Luminex technology and a mouse cytokine antibody bead kit (Milliplex map kit, Millipore) according to the manufacturer's specifications.

## Supporting Information

Figure S1 **Infection efficiency and cellular tropism within mice lungs, in the presence or absence of SARS-CoV E protein IC activity.** 16 week-old BALB/c mice were infected with 100000 PFU of the parental virus (wt, black columns) displaying E protein IC activity or the mutant virus lacking IC activity N15A (red columns). At 2 and 4 dpi mice were sacrificed and their lungs were fixed in formalin, paraffin embedded, sectioned and processed for immunofluorescence. SARS-CoV N protein and cell nuclei were labeled to discriminate both non-infected and infected cells. The number of alveolar (alveo), bronchiolar (bronch) and overall infected cells (total) were calculated in several representative images, and represented as percentages of their corresponding total cells (infected plus non-infected). Error bars indicate the standard deviation from the data collected from different images.
(TIF)
