# Supplementary figures and images for: Severe Acute Respiratory Syndrome Coronavirus Envelope Protein Ion Channel Activity Promotes Virus Fitness and Pathogenesis
Source: PLoS Pathog. 2014 May 1;10(5):e1004077. doi: 10.1371/journal.ppat.1004077 (PMC4006877; doi:10.1371/journal.ppat.1004077)

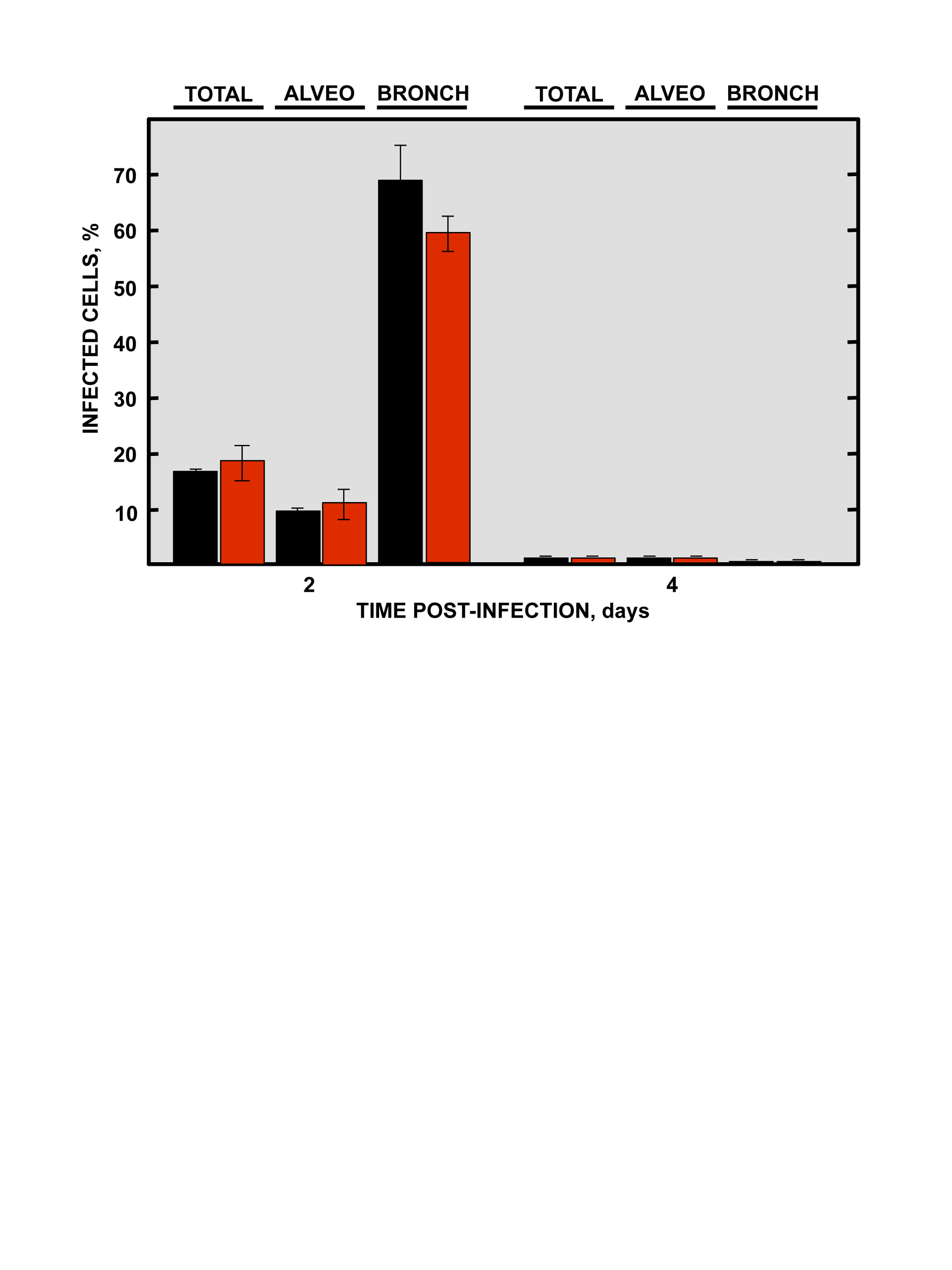

Supplement: Figure S1 — Infection efficiency and cellular tropism within mice lungs, in the presence or absence of SARS-CoV E protein IC activity. 16 week-old BALB/c mice were infected with 100000 PFU of the parental virus (wt, black columns) displaying E protein IC activity or the mutant virus lacking IC activity N15A (red columns). At 2 and 4 dpi mice were sacrificed and their lungs were fixed in formalin, paraffin embedded, sectioned and processed for immunofluorescence. SARS-CoV N protein and cell nuclei were labeled to discriminate both non-infected and infected cells. The number of alveolar (alveo), bronchiolar (bronch) and overall infected cells (total) were calculated in several representative images, and represented as percentages of their corresponding total cells (infected plus non-infected). Error bars indicate the standard deviation from the data collected from different images. (TIF) [file ppat.1004077.s001.tif]
